# Mechanistic Comparison of Semi-Solid Extrusion 3D-Printed Printlets and Hot-Moulded Tablets: Linking Polymer–API Interactions, Microstructure, and Dissolution of Plant-Based Formulations

**DOI:** 10.3390/pharmaceutics18081035

**Published:** 2026-08-20

**Authors:** Emilija Nemickaite, Pooja Todke, Vaidotas Cicenas, Elena Jasiūnienė, Mindaugas Marksa, Jurga Bernatoniene

**Affiliations:** 1 Department of Drug Technology and Social Pharmacy, Faculty of Pharmacy, Medical Academy, Lithuanian University of Health Sciences, Sukileliu pr. 13, LT-50161 Kaunas, Lithuania; emilija.nemickaite@lsmu.lt (E.N.); pooja.ashok.todke@lsmu.lt (P.T.); 2 Institute of Pharmaceutical Technologies, Faculty of Pharmacy, Medical Academy, Lithuanian University of Health Sciences, Sukileliu pr. 13, LT-50161 Kaunas, Lithuania; 3 Ultrasound Research Institute, Kaunas University of Technology, K. Barsausko Str. 59, LT-51423 Kaunas, Lithuania; vaidotas.cicenas@ktu.lt (V.C.); elena.jasiuniene@ktu.lt (E.J.); 4 Department of Electronics Engineering, Kaunas University of Technology, Studentu Str. 50, LT-51368 Kaunas, Lithuania; 5 Department of Analytical and Toxicological Chemistry, Medical Academy, Lithuanian University of Health Sciences, LT-50161 Kaunas, Lithuania; mindaugas.marksa@lsmu.lt

**Keywords:** molecular modelling, moulded tablet, printlets, hot moulding, semi-solid extrusion, release behaviour

## Abstract

**Background**: Three-dimensional printing (3DP) is rapidly advancing personalised medicine, yet systematic performance comparison with conventional manufacturing remains limited, particularly for plant-based formulations. **Methods**: This study compared tablets containing plant-based APIs (cannabidiol, apigenin, and luteolin) produced via conventional hot moulding and semi-solid extrusion (SSE) 3DP. The formulations were evaluated for physicochemical, mechanical, rheological, structural, and drug-release properties. **Results**: Both manufacturing methods produced tablets with comparable dimensions and mass; however, pronounced formulation-dependent differences were observed in mechanical strength, rheology, and microstructure. The molecular modelling predictions were consistent with the experimental findings. Agar–pectin exhibited the strongest predicted polymer–polymer and polymer–API interactions, including multiple hydrogen bonds, and formed a comparatively dense and cohesive matrix associated with slower API release. In contrast, the weaker interactions predicted for gelatine–pectin were associated with a less cohesive and more porous matrix that facilitated medium penetration, API diffusion, and drug release. SSE printlets generally exhibited greater porosity and more heterogeneous internal architectures than moulded tablets, resulting in enhanced drug release of approximately 95%. Micro-CT analysis provided important structural confirmation; API incorporation increased the void volume of gelatine–pectin printlets from 1.15% to 8.77%, demonstrating that disruption of polymer interactions contributed to pore formation and enhanced molecular diffusion. The observed release behaviour correlated with predicted molecular interactions and experimentally observed microstructural features, where increased porosity and weaker polymer–API interactions facilitated enhanced drug diffusion. **Conclusions**: Overall, SSE-3DP outperformed conventional moulding, demonstrating superior tunability and performance. This work provides a mechanistically informed strategy for designing plant-based, personalised natural products using 3DP technologies.

## 1. Introduction

Conventional manufacturing techniques like direct compression, dry granulation, and wet granulation have long been the norm for producing tablets because they provide accurate dosing, scalability, reproducibility, cost-effectiveness, and good patient compliance [1,2,3]. However, the concept of large-scale, centralised production using tablet presses has remained fundamentally unchanged for over a century. It curbs flexibility in dose individualisation and the incorporation of complex geometries or multiple release profiles, while also requiring proven long-term stability [4,5]. For regular tablets the most commonly used manufacturing techniques are via pressing and compression or via trituration, produced by forming a mass. Compressed tablets can be divided into direct compression of powder or tablet mixture, wet granulation and dry granulation. For soft, gummy, and gel-based dosage forms that are unsuitable for conventional compression, moulding provides a practical alternative. In hot moulding, a heated, flowable formulation is dispensed into predefined moulds and subsequently cooled or gelled to establish its shape and mechanical stability. Other approaches applicable to soft dosage forms include cold-set moulding, automated material deposition, continuous extrusion followed by cutting, and freeze-drying [6,7]. Although these traditional methods support reproducible and cost-effective large-scale production, their dependence on fixed tooling or mould geometry limits rapid adjustment of dose, shape, internal architecture, and drug-release behaviour, particularly for low-dose or sensitive active pharmaceutical ingredients (APIs) and formulations intended for paediatric, geriatric, or otherwise individualised therapy [8,9].

In contrast, three-dimensional (3D) printing has recently developed as a transformative approach in pharmaceutical manufacturing, providing control over tablet geometry, internal structure, and drug distribution [10,11]. The semi-solid extrusion (SSE) method facilitates layer-by-layer fabrication of tablets with adjustable doses and defined release profiles, allowing on-demand production and patient-specific formulations while reducing excipient loss [12]. By overcoming several challenges of conventional manufacturing, 3D printing offers a radical solution toward the development of personalised medicines. Nonetheless, systematic studies comparing traditionally manufactured with 3D-printed tablets (printlets) are required to evaluate their physical characteristics, mechanical strength, and drug-release profile, thereby providing the evidence needed to support clinical translation and regulatory approval.

An additional challenge arises when formulating printlets containing medicinal plant extracts, as these extracts differ from conventional pharmaceutical APIs because of their complex composition, hygroscopicity, and pronounced bitterness. Taste masking is particularly critical for ensuring patient acceptability, especially in paediatric and geriatric populations where palatability and ease of administration strongly influence compliance [12,13]. Traditional methods like coating, β-cyclodextrin/polysaccharides complexation, or the use of sweeteners often deliver partial masking and may reduce drug loading or affect stability [14,15]. SSE printing allows precise loading of bitter plant extracts into polymer matrices along with taste-masking agents, and enables the creation of porous internal structures that enable fast disintegration and rapid release. In contrast to conventional methods, SSE enables on-demand tailoring of dose, size, and flavour within a single process, facilitating the development of patient-friendly, plant-based dosage form with improved compliance and therapeutic outcomes.

Previous studies have widely examined the printlets containing pharmaceutical APIs such as omeprazole [16] and metformin [17]; however, only a few studies have focused on plant-based APIs [18]. This highlights an unmet need for the development of printlets containing plant-based APIs and emphasises the potential of SSE for producing age-specific, patient-centric printlets, importantly in low-resource settings. To date, no comparative framework has been published evaluating comparison of conventional tablets with printlets with respect to visual appearance, texture, and drug-release profiles, and limited studies are available on printlets containing plant extracts.

For the development of such systems, understanding formulation science becomes crucial, as the choice of polymers and their interactions with the drug directly influence performance. Therefore, the selection of optimal polymers is critical for tailoring drug release. Molecular interactions, particularly hydrogen bonding and ionic interactions between drug and polymer, play a crucial role in modulating dissolution and release behaviour. Benko et al. showed that hydrogen bond-based solid-state interactions, identified by FT-IR, significantly enhance drug retention and sustained release in implantable matrices [19]. Similarly, Que et al. reported that hydrogen bonding in amorphous solid dispersions reduces drug release, demonstrating its strong influence on dissolution kinetics [20]. In the case of ionic bonding, a cationic Salecan-g-PMAPTAC hydrogel exhibited a markedly sustained release of diclofenac due to strong ionic affinity, whereas insulin showed faster release [21]. Likewise, Salecan–PNM semi-IPN hydrogels also exhibited pronounced pH-responsive behaviour, with significantly higher DOX release at acidic pH (5.0) than at physiological pH (7.4), due to increased DOX protonation and weakened electrostatic interactions within the network [22]. This finding highlights the importance of assessing the molecular interactions to design the tablets with controlled and predictable drug-release profiles.

This work presents the first comprehensive investigation to test the hypothesis that modelling molecular interactions between polymers and plant-based APIs can help in predicting formulation behaviour and the differences in the release profiles for rationally designed moulded tablets and printlets. The present research also aims for a systematic comparison of conventional moulded tablets formulated through moulding and SSE 3D printing, with emphasis on an advanced structural analysis and drug-release behaviour. Furthermore, the study also explores the incorporation of *Artemisia annua* L. extract (phytochemicals such as apigenin and luteolin) and cannabidiol (CBD) into SSE-printlets, focusing on the role of different excipient combinations. The insights gained provide a novel molecular–mechanistic correlation, offering a rational design pathway for next-generation, nature-inspired 3D-printed drug delivery systems.

## 2. Materials and Methods

### 2.1. Materials

The dried *Artemisia annua* L. herbs were purchased from “Pamario žolynai” (Klaipėda, Lithuania). The chemicals used for this research were crystal CBD purchased from UAB Bioremedium (Vilnius, Lithuania), 96% ethanol (Vilnius, Lithuania), L-Glutathione ≥ 98% (ROTH, Karlsruhe, Germany), gelatine (molecular weight (MW) 50–100 kDa) and citric acid purchased from Sigma-Aldrich (Steinheim, Germany), agar (MW 175 kDa) purchased from Alvo (Panevėžys, Lithuania), pectin (MW 200 kDa) from citrus purchased from TCI (Zwijndrecht, Belgium), hydroxypropyl-methylcellulose (HPMC) “Methocel K15M” (viscosity of 15,000 cP, MW 575 kDa) purchased from IFF (New York, NY, USA), and sugar purchased from AB Nordic Sugar (Kėdainiai, Lithuania).

### 2.2. In Silico Study

#### 2.2.1. Generation of the Chemical Structures of API and Excipients

**Figure 1:**
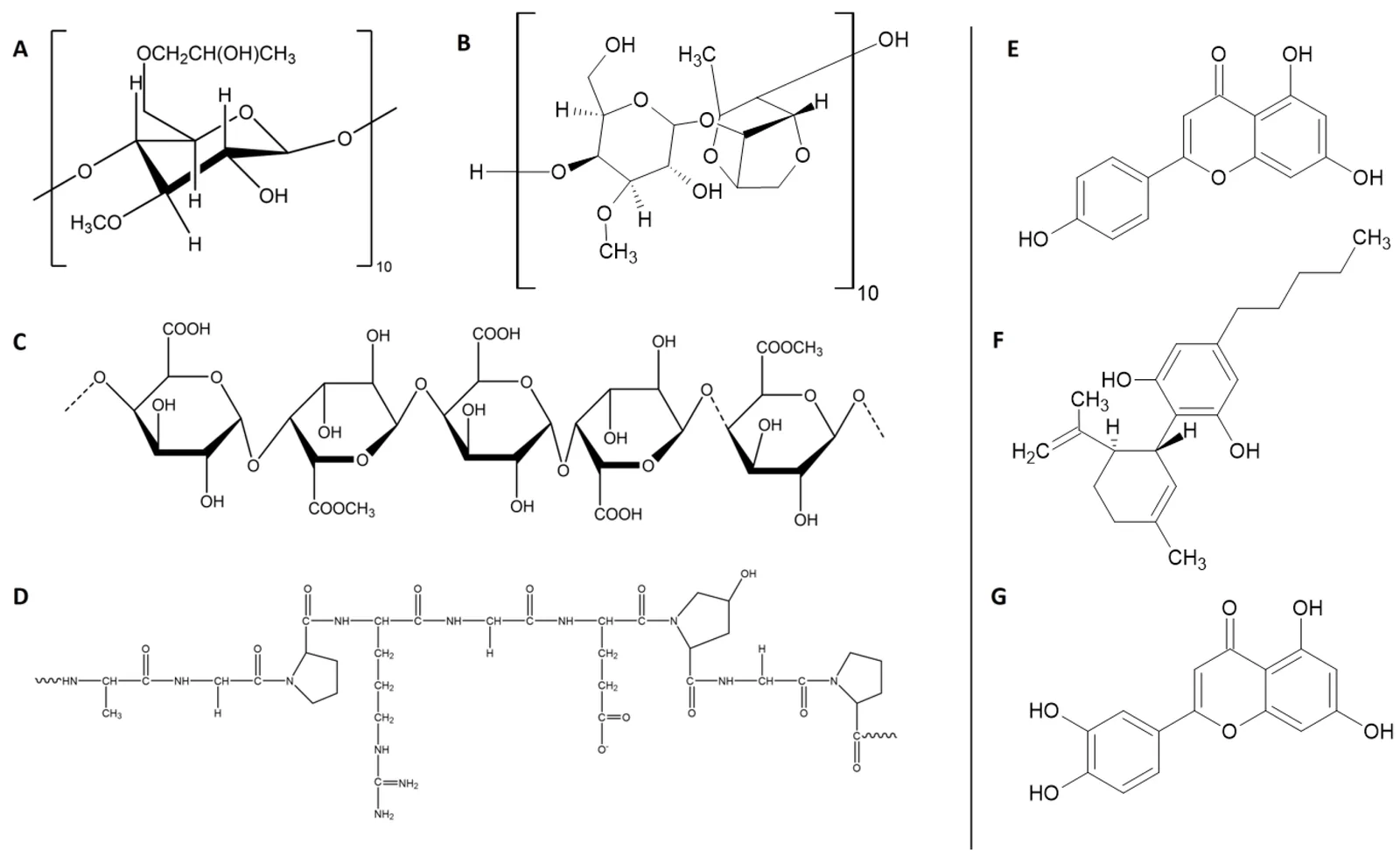
Representative chemical structures used in the molecular modelling: (**A**) HPMC oligomer (*n* = 10); (**B**) agar oligomer (*n* = 10); (**C**) pectin; (**D**) gelatine; (**E**) apigenin; (**F**) cannabidiol (CBD); and (**G**) luteolin.

The 3D structures of apigenin, CBD, and luteolin were retrieved from the ChemSpider database, and energy minimization was performed prior to docking [23]. The chemical structures of excipients including agar, HPMC, gelatine, and pectin were generated using ChemDraw^®^ Ultra version 10 (Cambridgesoft, Waltham, MA, USA). Polymers were represented using short oligomeric chains to ensure computational feasibility. HPMC was modelled as an oligomer containing 10 substituted anhydroglucose units (*n* = 10), while agar was represented by an oligomer containing 10 agarose-type disaccharide repeating units (*n* = 10). No fixed number of repeating units was assigned to gelatine or pectin because of their structural heterogeneity. Instead, gelatine was represented by a defined collagen-derived peptide segment, and pectin was represented by a defined galacturonic-acid-based polysaccharide segment. Initially, energy minimization of all polymers and APIs was carried out using the software package Materials Studio (version 2017, Accelrys Inc., San Diego, CA, USA) followed by geometry optimization employing Forcite module using COMPASSII force field. The representative chemical structures used in the molecular modelling are shown in Figure 1.

#### 2.2.2. Molecular Modelling

Molecular interactions between polymers were studied using Molecular Operating Environment (MOE, version 2022.01, Montreal, QC, Canada). Partial atomic charges were calculated, and energy minimization was performed using the steepest-descent protocol with the Merck Molecular Force Field (MMFF94X) at a root mean square (RMS) gradient of 0.0001 Å. Polymer–polymer docking was conducted at a 1:1 molar ratio, where one molecule of polymer was docked against one molecule of polymer 2 using the induced-fit protocol. Docking scores, calculated using the London dG scoring function, were further refined by two independent runs of the Triangular Matcher method. Binding poses were ranked using a consensus scoring approach combining London dG and GBVI/WSA dG scoring functions. The lowest binding energy conformations were selected and analysed visually. Graphical representations were generated in MOE [24]. Following this, the resulting polymer–polymer complexes were further modelled with each (apigenin, CBD, and luteolin) using the same docking protocol as described above.

### 2.3. The Extraction Process of Artemisia annua L. Plant

The dried herb of the *Artemisia annua* L. was milled using a trapezoid 0.5 mm hole sieve at 800 rpm using the Ultra Centrifugal Mill ZM 200 (Retsch, Haan, Germany). The ground plant material was soaked in 80% ethanol (*v*/*v*) and 0.25 mg L-glutathione. L-glutathione was selected based on our previous studies, which evaluated several extraction excipients, including titanium dioxide, β-cyclodextrin, propylene glycol, and L-glutathione. HPLC analysis demonstrated that L-glutathione provided the highest extraction yield of phytochemical compounds in both *Artemisia annua* L. and *Artemisia vulgaris* L [25]. The sample was later sonicated in the ultrasound water bath “Grant XUB10” (Grant Instruments, Cambridge, UK) and centrifuged at 3000 rpm for 15 min using “Sigma 3-18KS” (SIGMA Laborzentrifugen, Os-terode am Harz, Germany). The obtained extract was dealcoholized down to 20% ethanol (*v*/*v*) extract.

### 2.4. Design and Development of Moulded Tablet and Printlets

Eight different types of moulded tablet and printlet formulations were prepared using four distinct polymeric combinations, both with and without the APIs, namely *Artemisia annua* L. extract and CBD powder. The amounts of the materials (*w*/*w* %) used are given in Table 1. Certain formulations were developed based on previously published methodologies with slight modifications [26,27,28]. For each formulation, purified water was divided into specific proportions to ensure effective dissolution of all the components. Gelatine was firstly soaked in 20% (*w*/*w*) purified water and let to swell for 10 min. After 10 min it was poured into a glass beaker, and an additional 10% (*w*/*w*) of purified water was added and heated up to 80 °C while continuously stirring at 800 rpm with a magnetic stirrer. The same preparation process was applied to agar. In a separate glass beaker, 20% (*w*/*w*) of water was heated up to 80 °C and kept stirring at 800 rpm; afterwards, the HPMC powder was gradually added to avoid clumps while making the gel. Pectin was also gradually added to a separate glass beaker, with 10% (*w*/*w*) purified water heated to 80 °C and stirred constantly at 800 rpm. Both HPMC and pectin mixtures were stirred for an additional 10 min to form homogeneous solutions. For all the moulded tablet and printlet formulations, the sugar syrup was prepared as follows: the citric acid and sugar were added to a glass beaker and dissolved in 10% (*w*/*w*) purified water to form a syrup-like mixture, and the solution was heated to 80° ± 2 °C and stirred at 800 rpm. The remaining water was added at the end after all the materials were incorporated.

The mixtures F1–F4 are control groups, while mixtures F5–F8 are experimental groups with APIs, being the *Artemisia annua* L. plant extract and CBD. For the mixture F1 the order of the components added was gelatine, pectin and syrup. The plant extract and CBD for F5 were added before pectin and syrup. A similar order applies to F2 and F6, with agar being the first component. The order of the components added to the formulation F3 was gelatine, syrup and HPMC gel. The plant extract and CBD for F7 were added before syrup and HPMC gel. The order of the components added to the formulation F4 was HPMC gel, pectin and syrup. The plant extract and CBD for F8 were added before pectin and syrup. The mixtures were kept warm and stirred continuously until all the materials were properly incorporated. Later the mixtures were used to prepare solid pharmaceutical forms in two separate technologies: the moulded tablet using traditional hot moulding, and printlets using semi-solid extrusion.

#### 2.4.1. The Hot-Moulded Tablet Technology

The heated mixtures at 80 °C were poured into square-shape moulds to form traditional moulded tablets. Once cooled to room temperature the moulds were stored in the refrigerator for 24 h, until solid shapes were formed.

#### 2.4.2. The 3D Printing Semi-Solid Extrusion Process

The 3D-printing process was done on a FABRX M3DIMAKER 1 (FABRX, London, UK) printlet 3D printer, and the shapes of the printlets were modified with Repetier-Host V2.3.1 software (Hot-World GmbH & Co. KG, Willich, Germany) using digital 3D STL files. The printing conditions were set on both the 3D printer and on Slis3r Slicer version V1.3.0 software (GNU General Public License). The formulations were poured into the 20 mL syringes and let to cool to room temperature. The printing conditions for the shapes were as follows: the infill density was set at 70% with the layer height at 0.8 mm. Once cooled the syringe was mounted in the printer and printed at 3 mm/s speed, extrusion width 2 mm and at 5 mm/s flow rate, maintaining 40 ± 2 °C temperature, except for mixtures F3 and F7, which were printed at 25 ± 2 °C. The syringe used had a 20 mL volume with a 15G (1.3 mm)-size nozzle. Once printed the printlets were stored in sealed containers in a cool and dry place, at room temperature. The printlets were printed in square shapes.

### 2.5. Swelling Degree Test of Different Polymers

A swelling degree test was performed to observe how each of the polymers swell in contact with distilled water. The swelling degree was determined gravimetrically. The known amounts of each dry polymer (gelatine, agar, HPMC and pectin) were accurately weighed (W_0_) and were placed in a fine mesh and immersed into 100 mL of distilled water at 25 ± 2 °C. The samples were removed from the water at specific time intervals of 5, 10, 20, 30 and 60 min, then gently put on the filter paper to remove the excess surface fluid and weighed immediately (W_t_).

The swelling degree (SD) was calculated as the percentage of increase in the sample mass relative to the initial dry mass using the following equation:

$$SD\left(\%\right)=\frac{W_{t}-W_{0}}{W_{0}}\;\times \;100$$

W_0_—initial dry weight of the polymer sample;

W_t_—the weight of the swollen sample at time (t).

Measurements were performed in triplicate, and the results were expressed in percentage, as mean ± standard deviation.

### 2.6. Viscosity Measurement of Polymeric Mixtures

The viscosity of the mixtures was measured using the rotational viscosimeter Alfa L series (Fungilab, Barcelona, Spain) equipped with an L4 spindle (length 115 mm, diameter 4 mm). The hot sample was poured into a tall thin tube and placed under the spindle, and the viscosity was measured at 100 rpm rotational speed, at 40 °C, and the measurements were taken after 15 s. The HPMC–gelatine mixtures (F3 and F7) were measured both at 20 °C and 40 °C. Results are provided in mPa∙s.

### 2.7. The Uniformity of Mass of Moulded Tablets and Printlets

Following the European Pharmacopoeia article “Uniformity of Mass of Single-Dose Preparations” (Eur. Ph.2.9.5.), the test analysis was conducted for both moulded tablets and printlets. Twenty samples of each formulation, F1–F8, were individually weighed on an analytical balance scale (KERN ABT 120-5DNM, KERN & SOHN GmbH, Balingen, Germany), and the mass was calculated in regards to the acceptable deviation range of ±5%.

### 2.8. Moisture Analysis of the Moulded Tablets and Printlets

Moisture content was measured on a DBS moisture analyser (KERN & SOHN GmbH, Balingen, Germany). Conventional moulded tablets and printlets were weighed and dried at 105 °C until complete moisture evaporation and until the weight remained constant. The test was repeated three times, and the average of the results was calculated expressed as a percentage (%).

### 2.9. Texture Analysis of the Moulded Tablets and Printlets

Hardness and stickiness of the tablets were measured with the texture analyser TA.XT Plus (Stable Micro Systems, Godalming, UK). Five freshly made moulded tablets and printlets of each formulation were placed on a plate and pressed with the flat base attached to the device descended, then returned to its starting position. The test was repeated five times for each series, and the program automatically calculated the average and created a graph of the results. The parameters for the analysis were pre-test speed 1 mm/s, test speed 2 mm/s, post-test speed 10 mm/s, distance 5 mm, and trigger force 0.049 N. The hardness and stickiness were measured in Newton (N) units.

### 2.10. Disintegration Tests of Moulded Tablets and Printlets

Disintegration tests were conducted according to the European Pharmacopoeia article (Eur. Ph 2.9.1.). Disintegration tests were done in the SOTAX CH-4147 DT2 (Aesch, Switzerland) apparatus at 37 ± 0.2° C. Each of the F1–F8 moulded tablet and printlet samples were put in the 6 tubes of the basket and operated in the two following mediums until fully disintegrated: the simulated gastric medium (pH 1.2) and the phosphate buffer (pH 6.8). The time of the disintegration was measured for each sample. The simulated gastric medium and the phosphate buffer were prepared according to the European Pharmacopoeia article (Eur. Ph 5.17.1). The time for the disintegration test was set at intervals of 30 min. After 30 min the tablets were inspected, if they were completely disintegrated or not.

### 2.11. In Vitro Dissolution of Moulded Tablets and Printlets

Dissolution tests were conducted according to the European Pharmacopoeia article (Eur. Ph 2.9.3). In vitro release of apigenin, CBD and luteolin was evaluated using the USP type II paddle apparatus (SOTAX CH-4147 AT 7 smart (Aesch, Switzerland)). The temperature was kept at 37 ± 0.5 °C with a paddle rpm of 50, and 400 mL dissolution medium was used. Dissolution media selected was simulated gastric medium pH 1.2 and intestinal fluid pH 6.8. The moulded tablets and printlets were placed in the basket stirring element and were later immersed in the apparatus cylinder with the simulated gastric medium. After 90 min the simulated intestinal fluid was added into the cylinder containing the moulded tablets and printlets. The samples were collected within the intervals of 60 min and filtered through the 0.45 μm membrane filter. The samples were both collected from the gastric environment and the intestine environment, within intervals of 60 min, and filtered through the 0.45 μm membrane filter to achieve a clear solutions for the chromatography analysis. The quantitative analysis of flavonoids, apigenin and luteolin was performed with high-performance liquid chromatography (HPLC) (see Section 2.13) and the CBD with gas chromatography (GC) (see Section 2.14).

### 2.12. API Release Kinetic Models

The kinetics of in vitro API release from the moulded tablets and printlets were evaluated by applying mathematical models like zero and first order, Higuchi square root, and the Korsmeyer–Peppas equation.

### 2.13. High-Performance Liquid Chromatography Analysis of Apigenin and Luteolin

The predominant active compounds were identified using HPLC. The analysis was performed on a Waters 2695 chromatographic system equipped with a Waters 996 diode array detector (Milford, MA, USA) and an ACE 5C18 chromatography column (250 × 4.6 mm). Data were processed using Empower 3, Waters Chromatography Data Software Version 3.8.0. The eluent system consisted of 0.1% trifluoroacetic acid as eluent A and 100% acetonitrile as eluent B. The elution program was as follows: 5% to 15% B from 0 to 8 min, 15% to 20% B from 8 to 30 min, 20% to 40% B from 30 to 48 min, 40% to 50% B from 48 to 58 min, 50% to 50% B from 58 to 65 min, 50% to 95% B from 65 to 66 min, 95% to 95% B from 66 to 70 min, and 95% to 5% B from 70 to 71 min. The injection volume was 10 μL, the column temperature was maintained at 25◦C, and the mobile phase flow rate was 1 mL/min, with a total run time of 81 min. The absorption of apigenin was measured at 340 nm, while luteolin was measured at 350 nm. Quantification was performed using the external standard method. Calibration curves were constructed, with R^2^ values of 0.999383 for luteolin and 0.998872 for apigenin.

### 2.14. Gas Chromatography Analysis of CBD

Gas chromatography with a flame ionisation detector (FID) analysis was conducted on the Shimadzu GC-2010 Plus (Tokyo, Japan). An Rxi-5 MS capillary column (30 m length, 0.25 mm inner diameter, and film thickness of 0.25 μm) was utilised. The temperature was programmed starting at 80 °C for 1 min, then ramped up to 250 °C at a rate of 10 °C/min. It was further increased to 310 °C at a rate of 30 °C/min and held for 7 min. The total run time was 30 min. Helium was used as the carrier gas at a flow rate of 1 mL/min. The detector temperature was maintained at 330 °C. The injection mode was split (1:10), with an injection volume of 1 μL.

### 2.15. Microscopic Analysis with Optical and Scanning Electron Microscope (SEM) of Printlets

The surface morphology, porosity and overall visual of the printlet were examined using an SEM (S-3400N, Hitachi Science & Technology, Tokyo, Japan) at an accelerating voltage of 5.0 kV. The experimental group of gelatine–pectin printlets was mounted on a metallic sample holder. The chamber was evacuated, and images were captured at magnifications ranging from 25× to 1000× at room temperature in vacuum conditions. The texture and surface of the tablets was also captured on the optical microscope (Nikon Eclipse 50i, Nikon Co., Tokyo, Japan) at 4× magnification.

### 2.16. The Visual and Porosity Analysis of the Printlets with X-Ray Microtomography (μ-CT)

The visual examination of the 3D-printed layers, pores and their distribution was completed with X-ray μ-CT microtomography. Investigations were performed using a RayScan 250E X-ray 3D computer tomograph (RayScan Technologies GmbH, Germany). Measurements were carried out using a microfocus X-ray source with an output voltage ranging from 10 to 230kV. During the measurements the X-ray source irradiates the test object with a cone beam, and a two-dimensional (2D) image is recorded at the detector (Supplementary Figure S2). A photograph of the experimental setup used is shown in (Supplementary Figure S3). A flat panel detector with 2048 × 2048 pixels was used. During the measurement 2520 projections were acquired as the object was rotated. The projections were acquired with an integration time of 2 s, averaging 5 times, a voltage of 120 kV, and a current of 120 µA. The focal spot of the X-ray source used was 8 µm, while the resolution achieved is dependent on the size of the investigated test object, and the resulting voxel size was 16 µm. Dimensional analysis was carried out using Avizo for Industrial Inspection 9.70 (FEI SAS, Thermo Fisher Scientific Inc., Bordeaux, France.) software.

### 2.17. Stability Study of the Moulded Tablets and Printlets

The fresh moulded tablets and printlets were stored in the constant climatic chamber (BINDER KBF 260, Tuttlingen, Germany) at 25 °C and 60% relative humidity (RH) for 6 months according to the ICH guidelines (Q1A (R2)). Samples were stored in closable zip bags and were double bagged. The samples of printlets and moulded tablets were collected and monitored for API content, moisture, texture (hardness and stickiness) as well as the disintegration and dissolution analysis at time stamps of 1, 3 and 6 months.

### 2.18. Statistical Analysis

The data were presented as mean ± SD. Statistical analysis was performed using the Kruskal–Wallis test (a non-parametric alternative to one-way ANOVA) to evaluate differences among groups. For the stability test comparison, the Wilcoxon signed-rank test was used. The statistical analysis was performed using SPSS (V. 29.0.0.0, IBM, Chicago, IL, USA). The level of significance was valued at *p* < 0.05.

## 3. Results and Discussion

### 3.1. Molecular Modelling of Different Polymeric Pairs

Agar, pectin, HPMC, and gelatine were specifically chosen for the in silico study because they are among the most widely used and regulatory-accepted excipients for gel-based tablet formulations. Each represents a distinct class of polymer with different gelation and swelling mechanisms: agar (rigid thermoreversible polysaccharide gel), pectin (ion-sensitive polysaccharide), HPMC (hydrophilic cellulose derivative), and gelatine (biocompatible protein gel former) [29,30,31,32,33,34]. This miscellany allowed us to systematically evaluate polymer–polymer interactions across thermo-sensitive polysaccharide–ion-sensitive polysaccharide, polysaccharide–cellulose, and protein–polysaccharide/cellulose combinations. By focusing on these four, the study aimed to cover the most appropriate polymer pair that can enable complementary gelation properties, mechanical strength, and drug-release modulation, making them rational candidates for immediate-release gel-based tablet design. Furthermore, molecular modelling was employed to investigate the roles of API–polymer system interaction in the drug-release behaviour.

**Figure 2:**
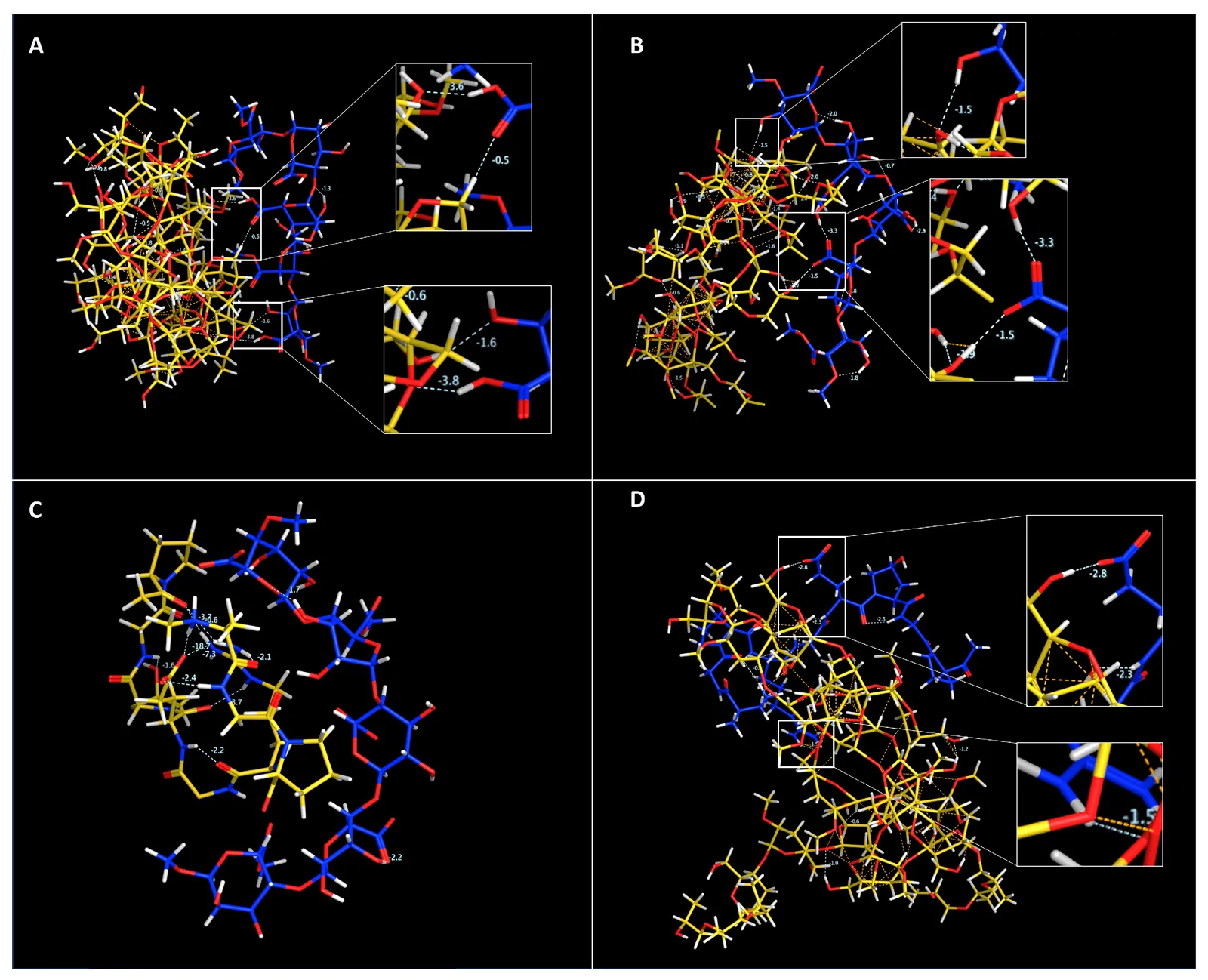
Molecular modelling of polymer pairs (Yellow—agar, HPMC and gelatine) (Blue—pectin and gelatine) 3D structures: (**A**)—agar–pectin, (**B**)—HPMC–pectin, (**C**)—gelatine–pectin and (**D**)—HPMC–gelatine systems. Oxygen and hydrogen atoms are shown in red and white, respectively.

The molecular modelling studies were carried out using the default docking protocol in MOE. Figure 2 depicts molecular modelling poses. The minimum binding energy and minimum hydrogen bonding energy confirmed maximum affinity between the polymer pairs. The number of hydrogens bonding was significantly higher with the agar–pectin pair compared with HPMC–pectin and HPMC–gelatine, while no H-bonding was observed for the gelatine–pectin pair (Table 2).

The agar–pectin pair exhibited the lowest binding energy (−8.72 kcal/mol), indicating strong molecular affinity that likely contributes to dense gel network with enhanced mechanical integrity and sustained release characteristics. The HPMC–gelatine pair also demonstrated a notable binding energy (−7.25 kcal/mol), reflecting good miscibility and suggesting a matrix with balanced elasticity, swelling behaviour, and structural stability. While HPMC–pectin (−6.32 kcal/mol) and gelatine–pectin (−6.23 kcal/mol) displayed weaker interactions in comparison (Table 2), the values remain sufficiently negative to justify their inclusion and potential utility in formulations where modulation of the release rate (faster release) or increased swelling is desired. Based on the molecular docking, we hypothesized that systems with stronger interactions may support sustained release, whereas weaker interactions may favour faster release.

### 3.2. Molecular Modelling of Polymer Complexes–APIs

**Figure 3:**
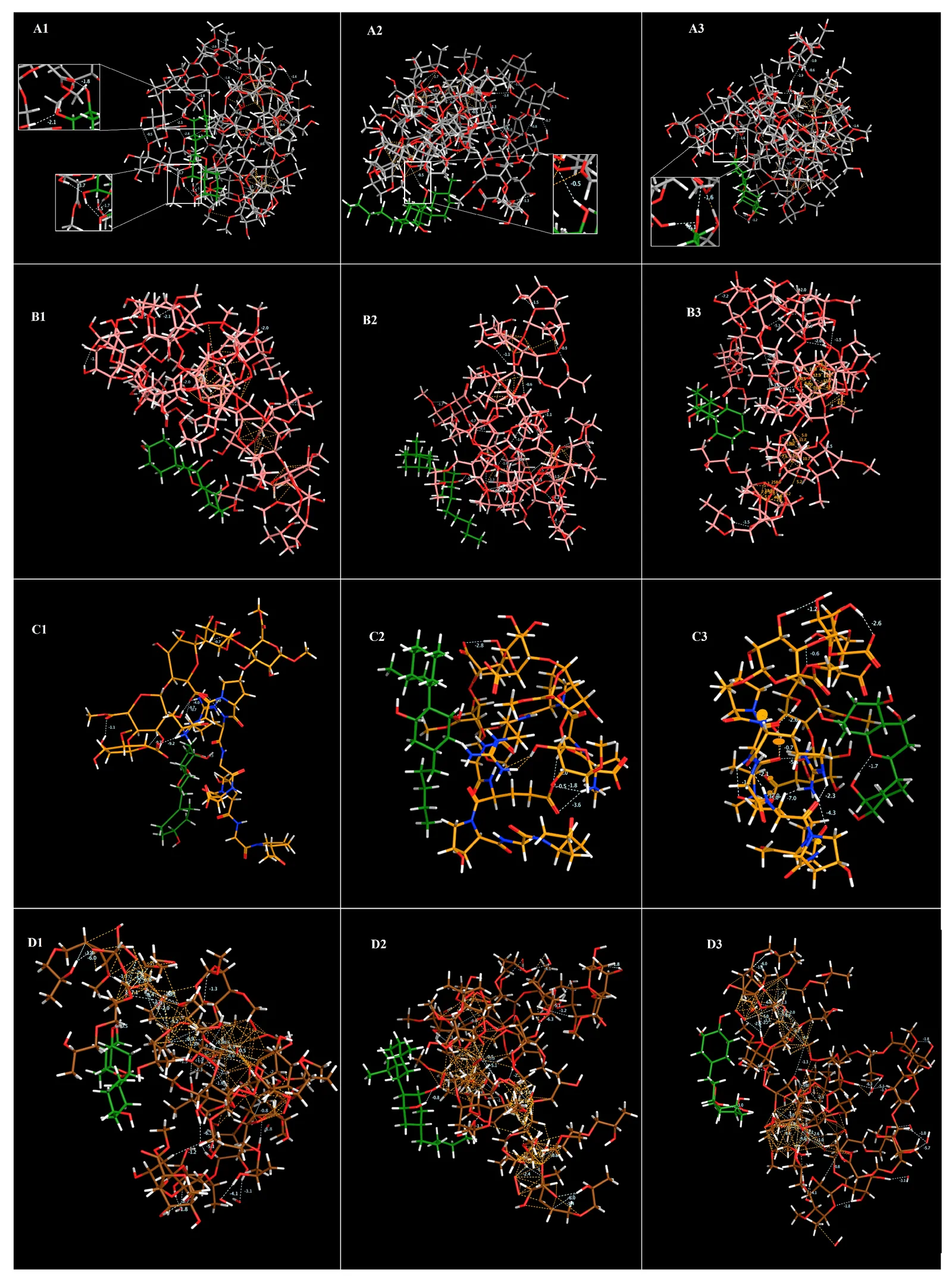
Molecular modelling of polymer complexes with APIs (API: green; polymer complexes—grey: agar–pectin complex; light pink: HPMC–pectin complex; yellow: gelatine–pectin complex; brown: HPMC–gelatine 3D structure); (**A1**): agar–pectin complex with apigenin, (**A2**): agar–pectin complex with CBD, (**A3**): agar–pectin complex with luteolin, (**B1**): HPMC–pectin complex with apigenin, (**B2**): HPMC–pectin complex with CBD, (**B3**): HPMC–pectin complex with luteolin, (**C1**): gelatine–pectin complex with apigenin, (**C2**): gelatine–pectin complex with CBD, (**C3**): gelatine–pectin complex with luteolin, (**D1**): HPMC–gelatine complex with apigenin, (**D2**): HPMC–gelatine complex with CBD, (**D3**): HPMC–gelatine complex with luteolin. Oxygen and hydrogen atoms are shown in red and white, respectively.

Molecular modelling of APIs including apigenin and luteolin of *Artemisia annua* L. extract and CBD with different polymer complexes (agar–pectin, HPMC–pectin, gelatine–pectin, and HPMC–gelatine) demonstrated negative binding energies, confirming favourable interactions. Figure 3 depicts molecular modelling poses. Among the modelled polymers pairs, the agar–pectin complex exhibited the strongest binding energies with hydrogen bonding with all three APIs (−5.81, −5.29, and −5.88 kcal/mol, respectively), suggesting a significantly higher affinity polymer–API system, which is expected to yield denser gel networks and slower drug diffusion, suggesting potential for sustained release. The HPMC–gelatine complex also displayed good binding affinity (−5.15 to −4.86 kcal/mol) with no H-bonding. Conversely, weaker or absent hydrogen bonding (e.g., gelatine–pectin) corresponded to lower binding affinities (−4.18 to –4.99) and looser networks, which may favour faster drug release (Table 3).

Thus, the extent of hydrogen bonding can be considered a key molecular element of release tailoring, where strong interactions propose sustained release, and weak interactions propose faster drug release.

### 3.3. Rheological Properties of Polymer Mixtures

The viscosity of the polymer mixtures was primarily evaluated at 40 °C; however, HPMC–gelatine formulations in both control and API-loaded polymeric mixtures were additionally analysed at 20 °C to reflect the actual printing conditions. HPMC–gelatine systems were printed at 20 ± 2 °C, as increasing the temperature to 40 ± 2 °C resulted in a transition to a low-viscosity liquid state, leading to poor shape fidelity and inadequate structural integrity during 3D printing. Despite this limitation, viscosity measurements at 40 ± 2 °C were conducted to enable direct comparison with pectin-based formulations.

**Figure 4:**
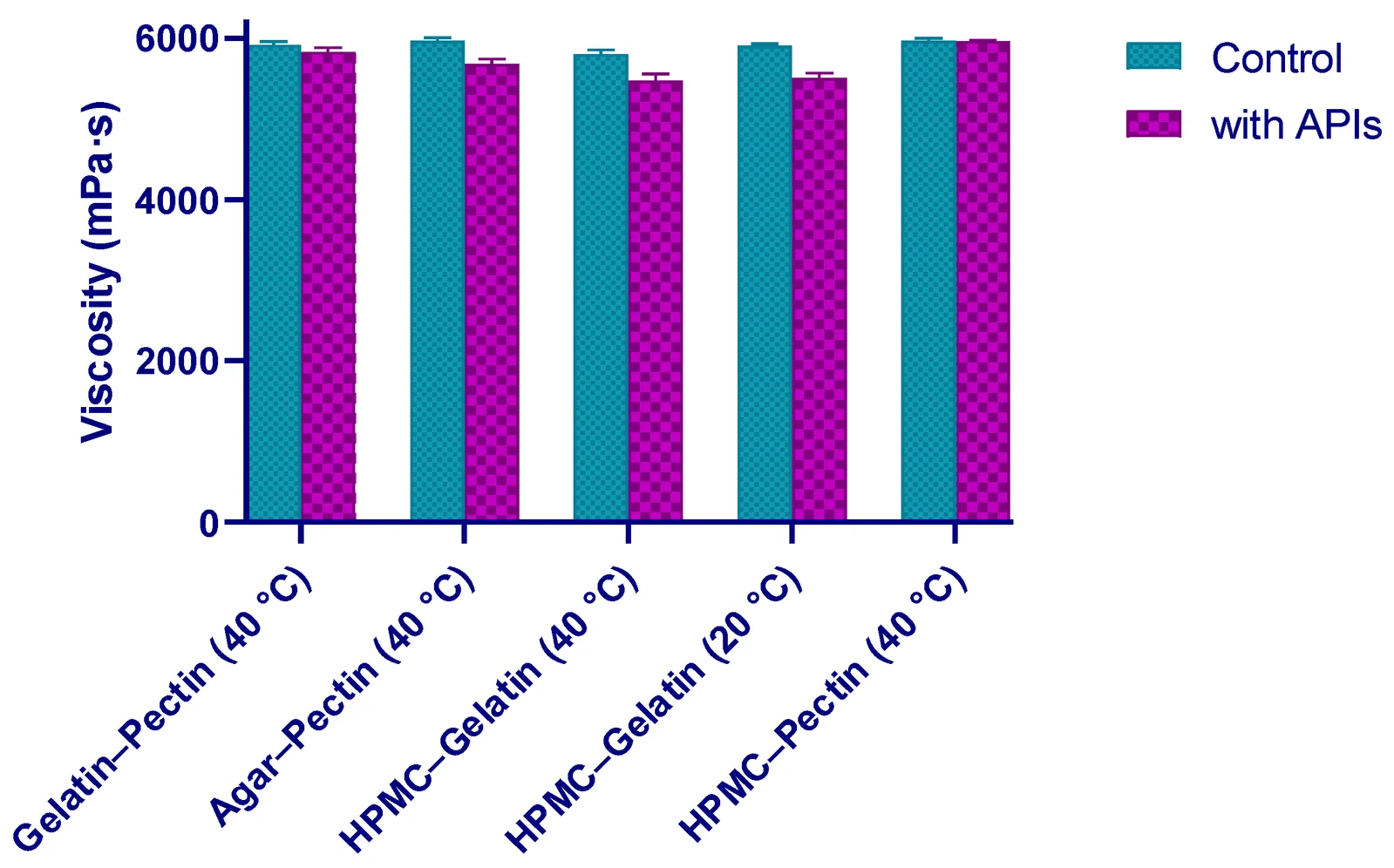
Viscosity of the polymer mixtures with APIs and without (control). Data is represented as mean ± SD, *n* = 3.

The viscosity values of the control and API-loaded mixtures are shown in Figure 4. Values ranged from 5475 ± 80.361 to 5972 ± 27.939 mPa∙s, with HPMC–pectin (control) showing the highest viscosity and HPMC–gelatine (with APIs) the lowest. Incorporation of APIs consistently reduced viscosity, suggesting that polymer–API interactions disrupted intermolecular networking within the polymer matrix. Multiple studies have demonstrated that APIs can reduce the viscosity of polymer gels through several, sometimes concurrent, mechanisms. Primarily, molecular docking and dynamics simulations revealed that APIs frequently form competitive hydrogen bonds with polymer chains, thereby disrupting the original polymer–polymer hydrogen bonding network that maintains gel cohesion and viscosity. By occupying hydrogen-bond acceptor/donor sites on the polymer, APIs weaken crosslink density, resulting in a less entangled and cohesive network [35,36,37]. Furthermore, API molecules may insert themselves between polymer chains, elevating chain mobility and decreasing chain entanglement, both known determinants of gel viscosity [38]. In thermosensitive gels, APIs can also alter micellar packing or increase intermicellar spacing, which further diminishes network integrity and viscosity [39].

Polymeric mixture containing HPMC–pectin and agar–pectin exhibited higher viscosities, while HPMC–gelatine-based systems with APIs showed lower values. These rheological behaviours are in good agreement with the molecular docking results. Stronger intermolecular networks were shown by the increased viscosities of the agar–pectin and HPMC–pectin systems, which were connected with higher binding affinities and multiple hydrogen bonds. Conversely, HPMC–gelatine with APIs compared to agar–pectin revealed lower binding affinities with no hydrogen bonding, which were correlated with faster gelation and lower viscosities. These results are supported by earlier studies that demonstrate that hydrogen bonding hinders polymer chain motion, which increases viscosity and has a major impact on the linear viscoelasticity and viscosity of entangled polymer melts [40,41]. Furthermore, in blends of poly (2-vinyl pyridine)/poly (4-vinyl phenol), the formation of hydrogen bonds was shown to increase the zero-shear viscosity and extend relaxation times, even though one polymer was below the entanglement threshold [42]. Similarly, concentrated poly(vinyl alcohol) solutions form hydrogen-bonded networks that yield gel-like structures with prolonged relaxation times, thereby affecting viscoelastic behaviour regardless of chain lengths [43]. These findings highlight hydrogen bonding as a critical determinant of viscosity and relaxation dynamics, and overall network characteristics in polymer systems. Thus, the molecular-level interactions between polymers and APIs provide a mechanistic basis for the observed differences in viscosity and gelation behaviour.

Moreover, the observed viscosity trends also correlate with the molecular weight of the polymeric pairs, which governs chain entanglement density and network formation. Gelatine, with a relatively low molecular weight (~100 kDa), when used alone exhibits low viscosity and minimal yield stress due to limited chain entanglement. However, when combined with polysaccharides such as pectin or sugars, its rheological performance improves significantly, as intermolecular interactions enhance structural cohesion and resistance to flow [26,44]. In contrast, HPMC (K15M grade), with a substantially higher molecular weight (~575 kDa) and an intrinsic viscosity of approximately 15,000 mPa·s, plays a dominant role in modulating formulation viscosity even at relatively low concentrations (as in F3, F4, F7, and F8; Table 1). Its long polymer chains promote extensive entanglement, thereby increasing resistance to deformation and flow. Similarly, polysaccharides such as agar (~175 kDa) and pectin (~200 kDa) contribute to viscosity enhancement through both molecular weight effects and their strong propensity for hydrogen bonding. Agar, in particular, forms rigid and brittle gel networks [45], while pectin facilitates cohesive matrix formation [46]. When combined, these polymers produce systems with higher effective molecular weights (e.g., agar–pectin ~375 kDa), leading to increased network density and viscosity [47,48].

Across the studied formulations, polymer pairings such as gelatine–pectin (~300 kDa), HPMC–gelatine (~675 kDa), and HPMC–pectin (~775 kDa) demonstrate a clear trend: higher combined molecular weight systems exhibit greater viscosity, reflecting enhanced chain entanglement and intermolecular interactions.

Overall, these findings reinforce that molecular weight, in conjunction with polymer–polymer interactions, is a key determinant of rheological behaviour. This directly influences critical performance attributes, including extrusion through the printing nozzle, shape retention, mechanical strength, and textural properties of the printed constructs.

### 3.4. Preparation of Moulded Tablets and Printlets

**Figure 5:**
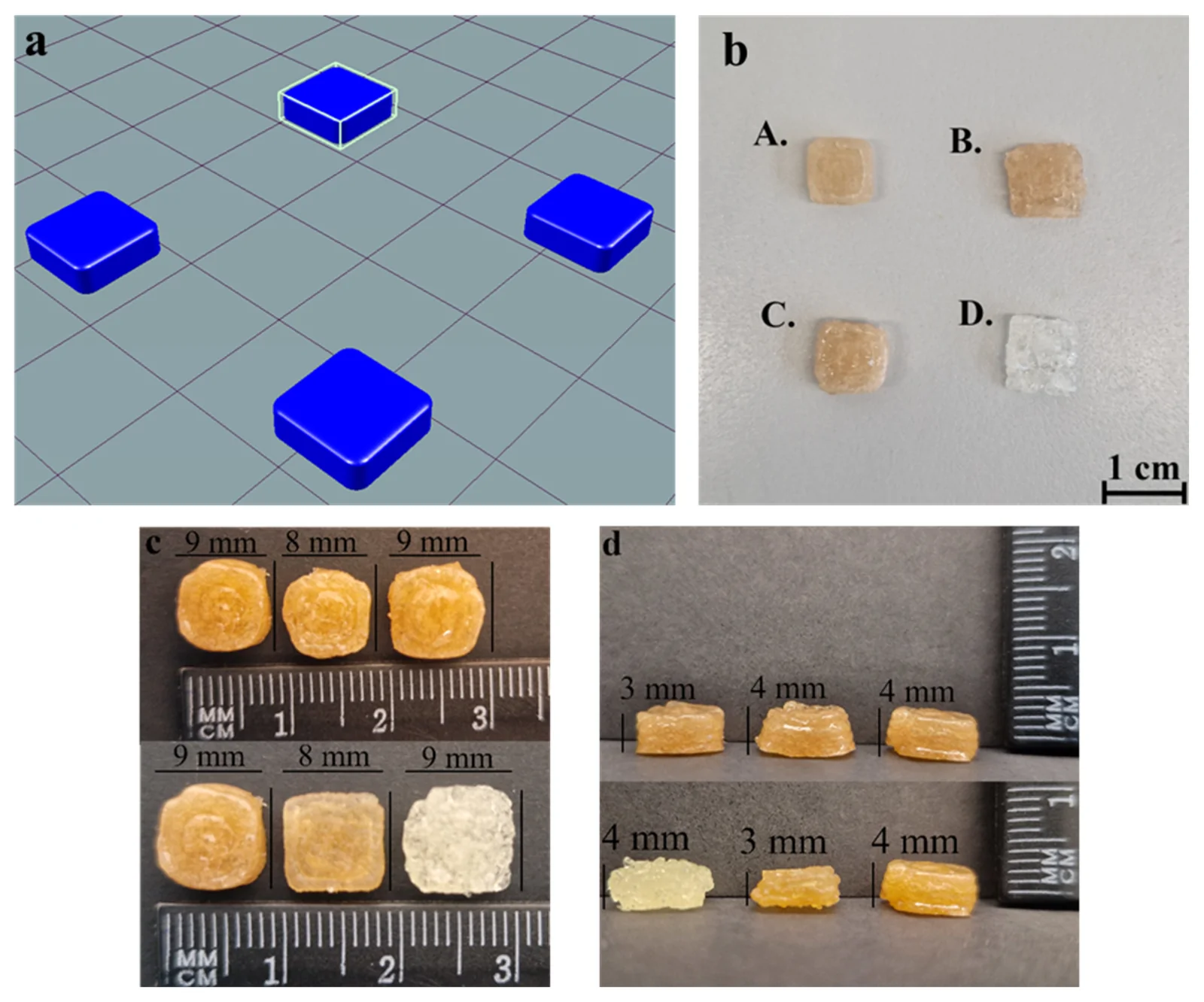
Visual representation of 3D-printed tablets. (**a**) STL file 3D visual. (**b**) 3D-printed tablets (printlets) with various polymeric mixtures combinations: A. agar–pectin, B. HPMC–pectin, C. gelatine–pectin and D. HPMC–gelatine. (**c**) Visual representation of the length and width (x and y dimensions) and their variation across replicates (*n* = 3). (**d**) Visual representation of the height (z dimension) and its variation across replicates (*n* = 3).

Moulded tablets and printlets containing *Artemisia annua* L. extract and CBD were prepared using both conventional moulding and SSE 3D printing, respectively. All eight polymer pair compositions produced tablets with consistent geometry with minor dimensional variations from the STL file dimensions; the standard deviation was statistically calculated (8.00 ± 0.78 × 8.00 ± 0.78 × 3.00 ± 0.14 mm (length × width × height), 0.8 mm layer height), demonstrating process reproducibility. Extrusion parameters, including pressure and feed rate, were adjusted for each formulation to ensure well-defined structures. The successful conversion of STL files into accurately printed geometries confirmed the reproducibility of the printing process (Figure 5a). The visual differences among the tablets prepared from different polymeric combinations (Figure 5b) further suggest that polymer composition significantly influences the surface integrity and print resolution.

Square tablets were chosen for further testing due to their consistent dimensions (8.00 ± 0.78 × 8.00 ± 0.78 × 3.00 ± 0.14 mm) and weight uniformity, with 5% deviation (Figure 5c,d). Among the tested polymer combinations, gelatine–pectin and HPMC–gelatine was more readily processed via moulding and printing, while agar–pectin and, particularly, HPMC–pectin posed greater fabrication challenges because of slower solidification. For agar–pectin and HPMC–pectin, solidification was completed within 24–48 h under refrigeration, compared with 2–3 h for gelatine–pectin and HPMC–gelatine. The observed differences in processability and solidification behaviour can be attributed to the intrinsic rheological properties of the individual excipients. Gelatine primarily exhibits thermally induced gelation and solvent phase transition [49,50], whereas agar gelation is driven by extensive hydrogen bonding [51]. HPMC demonstrates pronounced shear-thinning behaviour [52], while pectin exhibits both shear-thinning and thixotropic characteristics [53]. Notably, moulded tablets generally exhibited faster moisture loss, whereas printlets, due to their layered structure, retained more moisture, a factor influencing disintegration and API release.

**Figure 6:**
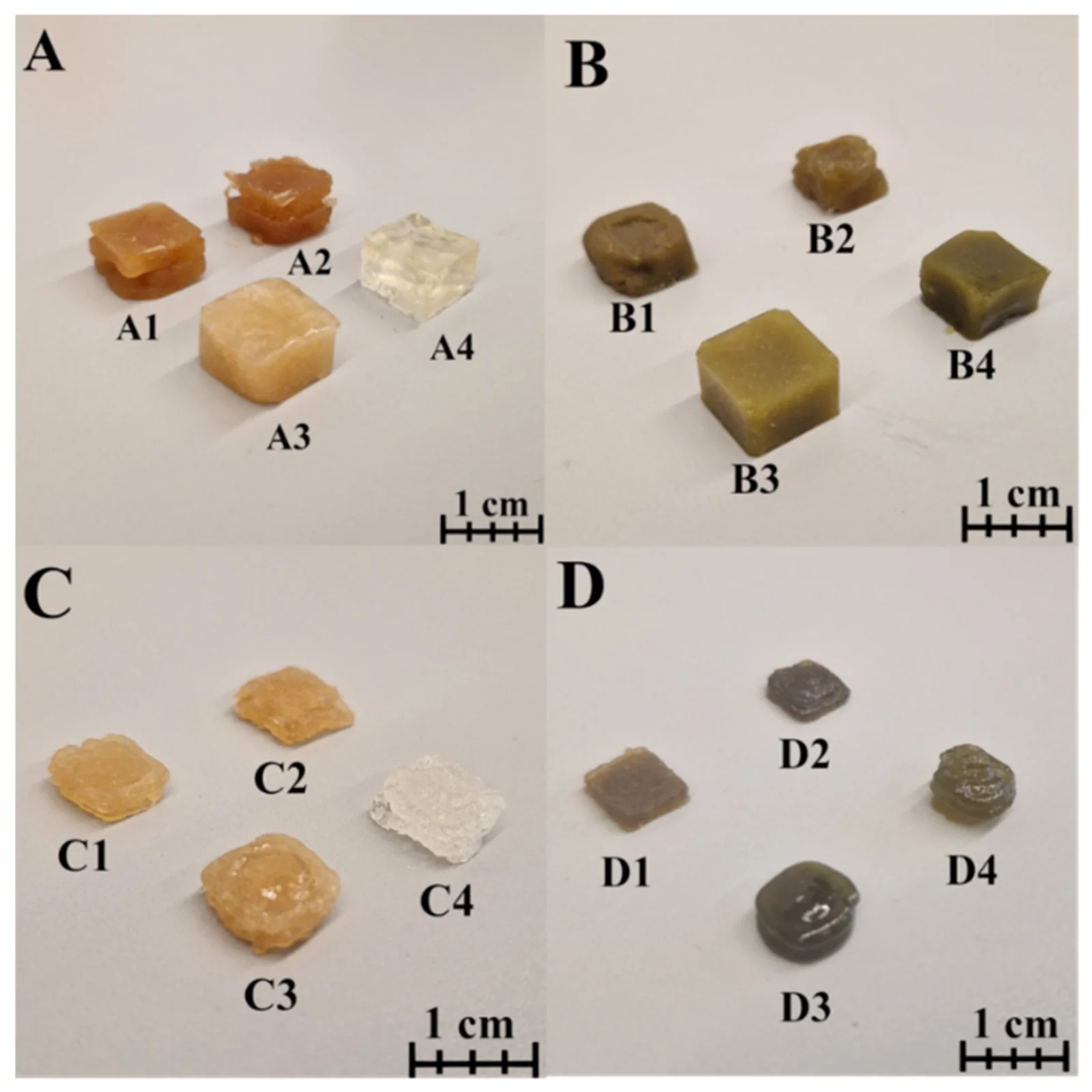
Visual comparison of moulded tablets and printlets prepared with different polymer pair combinations (arranged in the same order as Figure 2). (**A**) Control group moulded tablets: A1 (agar–pectin), A2 (HPMC–pectin), A3 (gelatine–pectin), A4 (HPMC–gelatine). (**B**) Experimental group moulded tablets (with APIs): B1 (agar–pectin), B2 (HPMC–pectin), B3 (gelatine–pectin), B4 (HPMC–gelatine). (**C**) Control group printlets: C1 (agar–pectin), C2 (HPMC–pectin), C3 (gelatine–pectin), C4 (HPMC–gelatine). (**D**) Experimental group printlets (with APIs): D1 (agar–pectin), D2 (HPMC–pectin), D3 (gelatine–pectin), D4 (HPMC–gelatine).

Clear visual differences were observed between placebo and API-loaded tablets. Placebo tablets (without A. annua extract and CBD) displayed pale shades of yellow, orange, or red (Figure 6A,C), while API-containing tablets were distinctly brownish-green due to phytochemical content (Figure 6B,D). Moulded tablets exhibited a softer, more uniform character, whereas printlets were firmer, slightly adhesive, and displayed a layered architecture absent in the moulded tablets (Figure 6). This structural layering is anticipated to affect texture, moisture retention, and drug-release performance.

Different combinations of the excipients enabled a clear difference not only in the 3D printing or the moulding process but also in the resulting texture, moisture and other characteristics. During the 3D-printing process, all formulations except for F3 and F7 were 3D printed at 25 ± 2 °C. Notably, F3 and F7 were the only formulations lacking pectin, suggesting its critical role in facilitating printability at elevated temperatures. As a polysaccharide, pectin contributes to gel network formation and enhances structural integrity at higher temperatures, thereby supporting shape fidelity during extrusion [54]. Likewise, HPMC–gelatine formulations also required a lower temperature of 25 ± 2 °C, as exposure to higher temperatures (30–40 °C) led to excessive liquefaction, resulting in droplet formation rather than well-defined structures. Although pectin-containing formulations could be processed at lower temperatures, this required substantially higher extrusion pressures due to increased viscosity, increasing the risk of nozzle clogging and compromising print stability and resolution [7,55].

Overall, SSE 3D printing facilitated the reproducible preparation of printlets with patient-adaptable design potential. The incorporation of *A. annua* extract and CBD imparted a characteristic brownish-green colour, contrasting with paler controls, providing a distinctive appearance that may enhance patient acceptance. Importantly, the printability trends observed across polymer pairs align with the molecular interaction study, where weaker polymer–polymer and polymer–API binding (e.g., gelatine–pectin) corresponded with lowest viscosity and faster gelation, influencing ease of fabrication and final tablet characteristics.

### 3.5. The Uniformity of Mass Test

The uniformity of mass was performed on both 20 samples of moulded tablets and 20 samples of printlets for each formulation (Table 4). Both moulded tablets and printlets had a mass deviation of ±5%, due to the mass being above 250 mg. The mass and its deviation were put into consideration while performing the other following tests.

While most printlets displayed a weight ranging from 0.80 ± 0.04 g to 0.86 ± 0.04 g, the moulded tablets weighed between 0.80 ± 0.04 g and 1.00 ± 0.05 g. This shows that printlets maintained a similar weight throughout the four formulations, whereas moulded tablets exhibited a certain mass difference. The 3D-printing process allows us to control not only the size and the shape of the drug more easily but also helps us maintain the same mass and dosage throughout all the formulations, with software that is quickly adjustable, more efficient and sustainable than using a simple moulding tray.

The difference may seem insignificant between moulded tablets and printlets at first, but the visual and the physical aspect of the two products, as well as the difference in the preparation technology, allows us to acknowledge the superiority of the 3D-printing process compared to the conventional preparation methods.

### 3.6. Swelling Degree of Polymers

**Figure 7:**
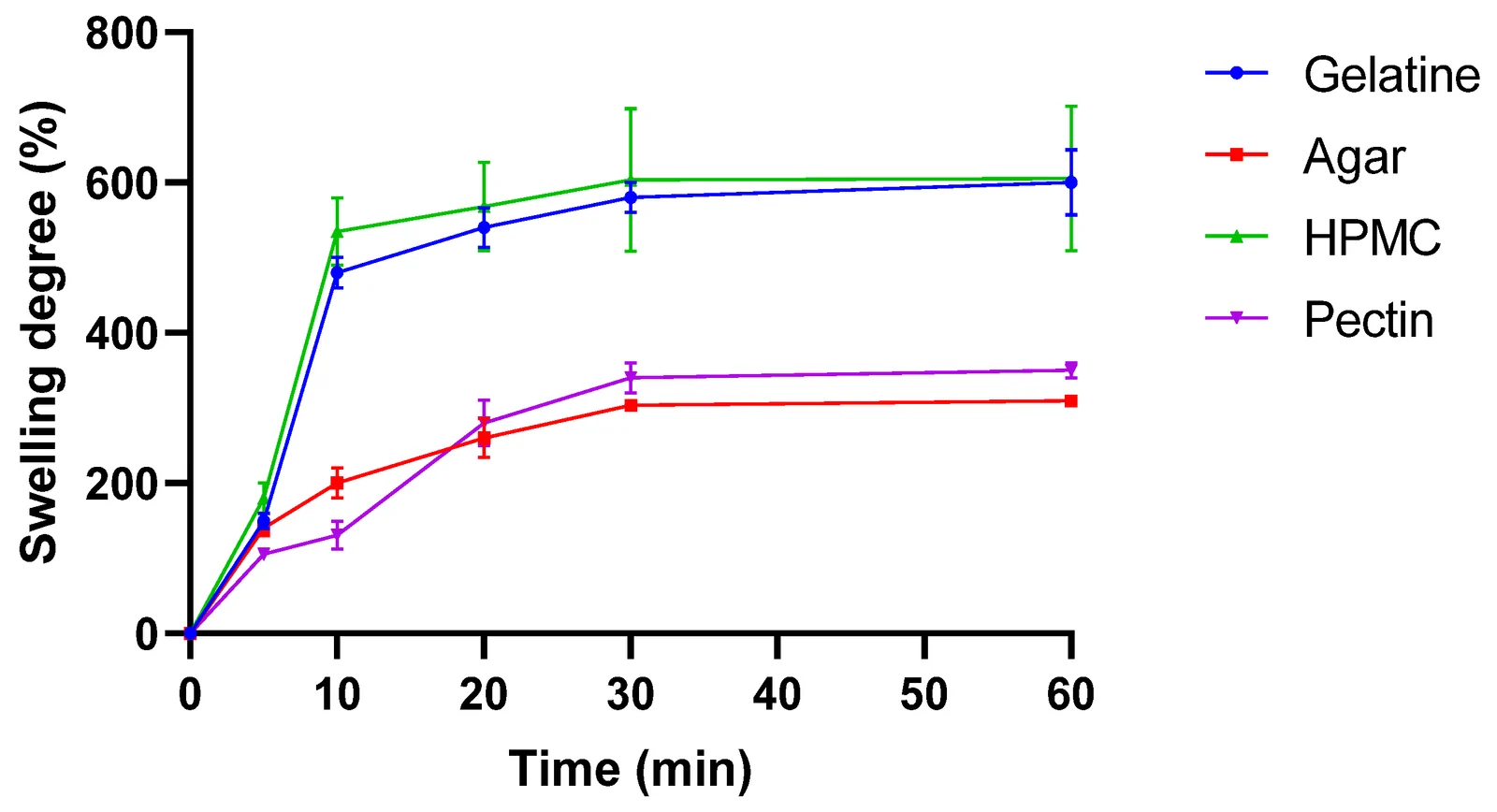
Graphical representation of each polymer’s swelling degree (%) over time.

The swelling test was completed within an hour, and all four polymers, namely gelatine, agar, HPMC and pectin, showed a significant mass growth when absorbed in water. The swelling degree of each polymer is presented in Figure 7.

It is observed that the highest swelling degree was present in HPMC (605% ± 18.5%) and gelatine (600% ± 17.3%). This further contributes to the fact that HPMC and gelatine have excellent gelling and swelling characteristics. However, the lowest swelling degree was present in agar (310% ± 4.8%), slightly lower than pectin (350% ± 11.5%). HPMC has high swellability characteristics, which is a key mechanism for controlling drug release in hydrophilic matrices. For gelatine, when it encounters water, its chains undergo relaxation, allowing water molecules to enter the network and soften the matrix. For agar, which is considered a strong gelling agent that acts as a swelling and viscosity-boosting agent in aqueous systems, in some formulations the water swelling saturation can be reached at approximately 102%, and pectin is a natural hydrocolloid with an excellent water absorption capacity, though its swelling degree is highly sensitive to the chemical environment, in this case distilled water (pH 5–6); pectin swells extensively as it transitions into a more soluble salt form [5,48,56,57].

According to our statistical analysis, we found that the swelling degree statistically significantly correlates to other assay characteristics except stickiness: with viscosity there was a moderate correlation (rs = 0.404, *p* < 0.05), with hardness the correlation was (rs = 0.301, *p* < 0.05), and there was a weak correlation with the moisture content (rs = 0.105, *p* < 0.05).

### 3.7. Moisture Analysis

**Figure 8:**
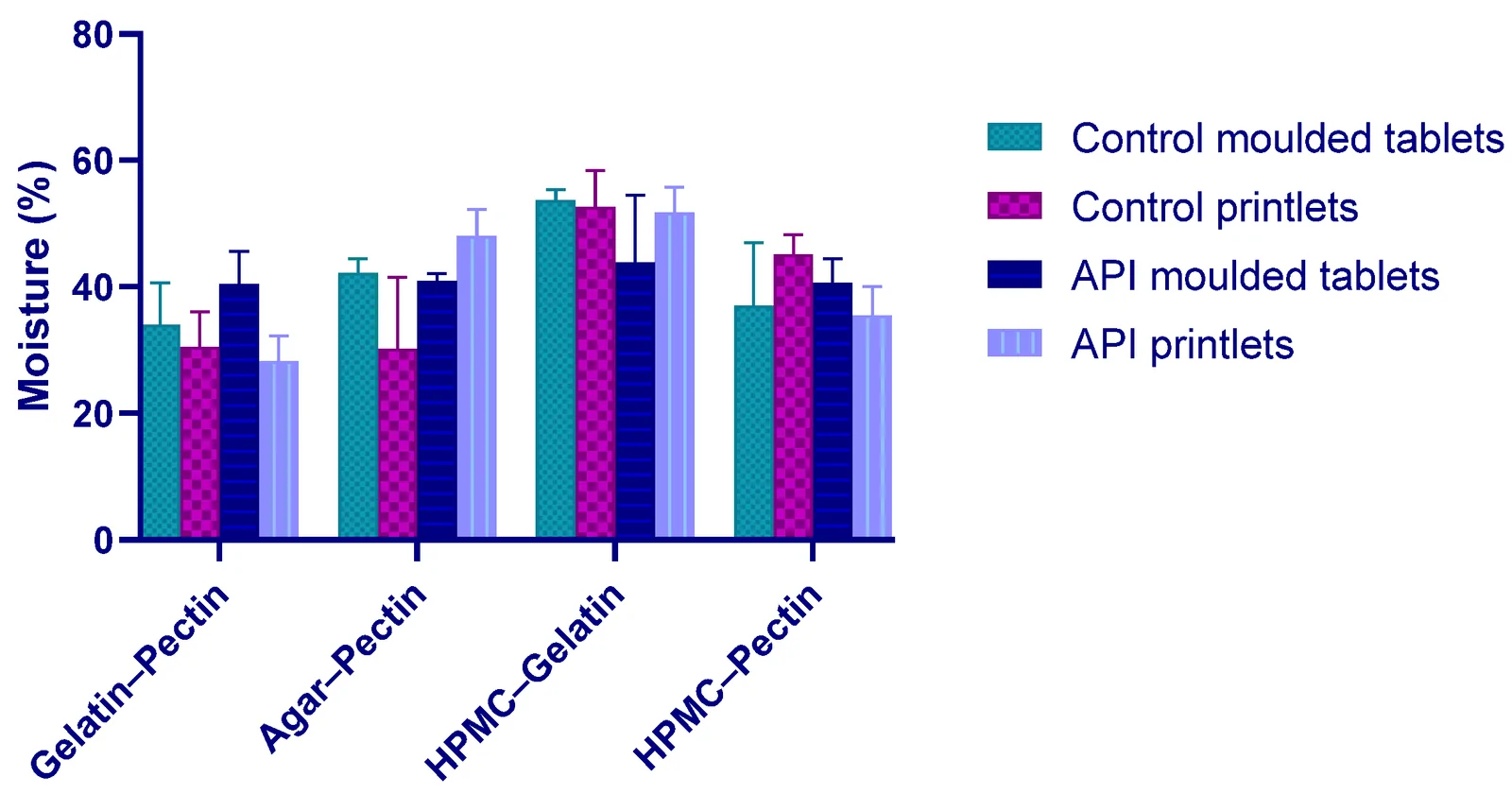
Moisture content of moulded tablets and printlets for both control and API-containing tablets. Data is represented as mean ± SD, *n* = 3.

The moisture content of solid dosage forms is a critical quality attribute, as it can directly affect their chemical, physicochemical, and microbial stability. Moisture of both moulded tablets and printlets was evaluated. For moulded tablets, moisture ranged from 34.0 ± 6.6% to 53.7 ± 1.7%, whereas printlets ranged from 28.3 ± 4.0% to 52.6 ± 5.8%. Among the controls, moulded gelatine–pectin tablets exhibited the lowest moisture (34.0 ± 6.6%), whereas printlets of gelatine–pectin (30.5 ± 5.5%) and agar–pectin (30.2 ± 11.3%) tablets showed similarly low levels (Figure 8).

The gelatine–pectin printlet in the experimental group containing APIs had the lowest moisture content (28.3 ± 4.0%, *p* > 0.05), indicating an efficient moisture reduction that is probably caused by pectin’s function during polymer swelling or blooming [58,59]. However, in line with HPMC’s hygroscopicity, HPMC–gelatine formulations (without pectin) demonstrated greater moisture retention (up to 51.8 ± 4.0% in printlets). Other formulations followed similar trends, e.g., HPMC–pectin control moulded tablets (37.0 ± 10.0%), experimental moulded tablets (40.6 ± 3.9%), and HPMC–pectin printlets with APIs (35.5 ± 4.5%) (Figure 8). Due to possible variations in drying procedures, the measured initial moisture levels are higher than the suggested maximum residual moisture for commercial gel tablets (≤5%). Over a few weeks at room temperature in sealed containers, the moisture content drops to ≤2%, highlighting effective long-term stability.

A moderate but statistically significant correlation was revealed between moisture content and viscosity (r = 0.260, *p* < 0.05), suggesting that higher-viscosity formulations tend to retain slightly more moisture [60,61]. The differences between moulded tablets and printlets are likely due to structural variations: the layered architecture of printlets may result in air pockets that influencing evaporation, disintegration, and drug release, whereas the more uniform moulded tablets lose moisture more quickly [10,11].

### 3.8. Hardness and Stickiness Texture Analysis

**Figure 9:**
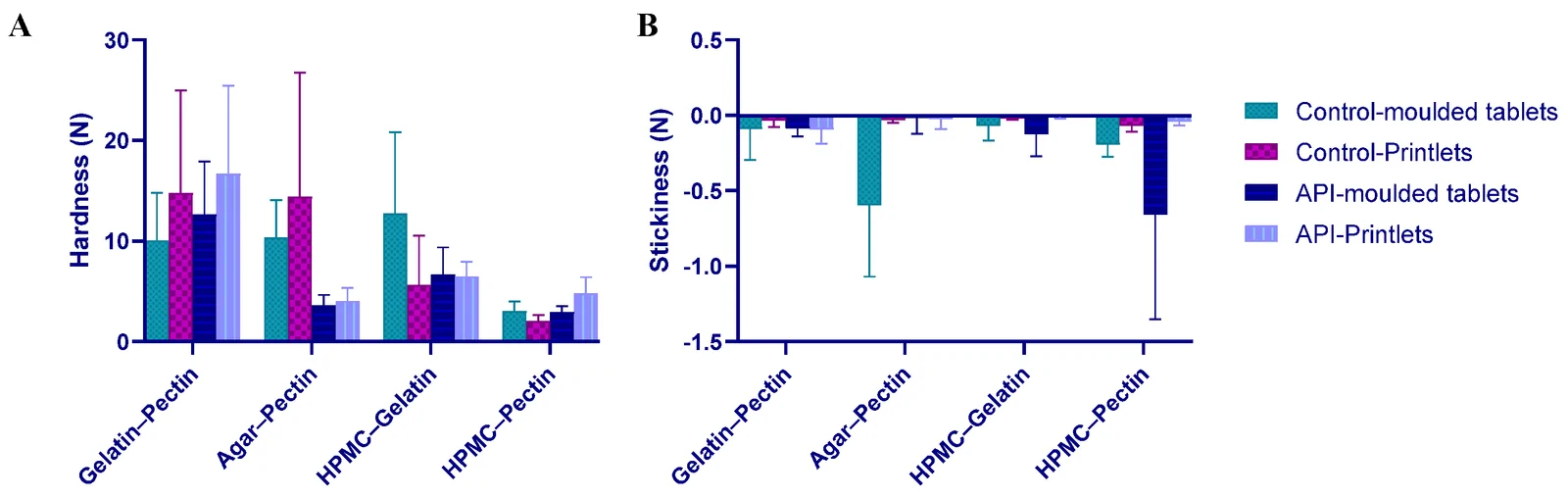
Texture analysis: Hardness (**A**) and Stickiness (**B**) of the moulded tablets and printlets for both control and experimental groups respectively. Data is represented as mean ± SD, *n* = 5.

The texture analysis revealed formulation- and process-dependent differences in hardness and stickiness between moulded tablets and printlets. Hardness in our case represents the strength of the gel under an applied force, mimicking the force applied by molar teeth during mastication. Values of hardness ranged from 2.028 ± 0.598 N to 16.700 ± 8.758 N, with gelatine–pectin printlets containing APIs showing the highest values (16.700 ± 8.758 N), highlighting their more compact and mechanically stable matrix. By contrast, HPMC–pectin tablets exhibited the weakest hardness, both as moulded tablet (3.060 ± 0.950 N) and printlet (2.028 ± 0.598 N) forms, despite having the highest viscosity. An increased HPMC concentration generally results in harder and more rigid tablets. As a cellulose derivative, increasing the concentration of HPMC enhances the absolute value of adhesiveness. However, HPMC concentration has a minimal effect on characteristics like cohesiveness and springiness [62,63]. In some gelatine/HPMC gummy formulations, HPMC incorporation had little effect on the final hardness of the product under chilled conditions [27,64,65]. In general, printlets improved hardness in gelatine–pectin and HPMC–pectin samples compared with moulded tablets (*p* < 0.05), underscoring the relevance of additive manufacturing for enhancing structural stability (Figure 9A).

Stickiness values were less uniform across formulations. The highest stickiness was observed for HPMC–pectin experimental moulded tablets (−0.658 ± 0.691 N) and gelatine–pectin printlets with APIs (−0.092 ± 0.098 N). The lowest stickiness occurred in HPMC–gelatine printlets (−0.015 ± 0.007 N) and moulded agar–pectin (−0.018 ± 0.104 N) tablets, showing the dependence of adhesive properties on both excipient interactions and the fabrication process. Interestingly, agar–pectin moulded controls also displayed elevated stickiness (−0.595 ± 0.475 N) not replicated in printlets (Figure 9B).

Weak but significant correlations existed between viscosity and hardness (rs = 0.264, *p* < 0.05) and between hardness and stickiness (rs = 0.153, *p* < 0.05), while viscosity did not correlate with stickiness. Although HPMC–pectin produced viscous dispersions, their gels lacked mechanical strength, suggesting polymer compatibility governs textural performance more than viscosity alone. Gelatine–pectin emerged as the most promising excipient blend for SSE 3D printing, combining high hardness with manageable stickiness.

### 3.9. The Optical Microscope and SEM Analysis of Printlets

**Figure 10:**
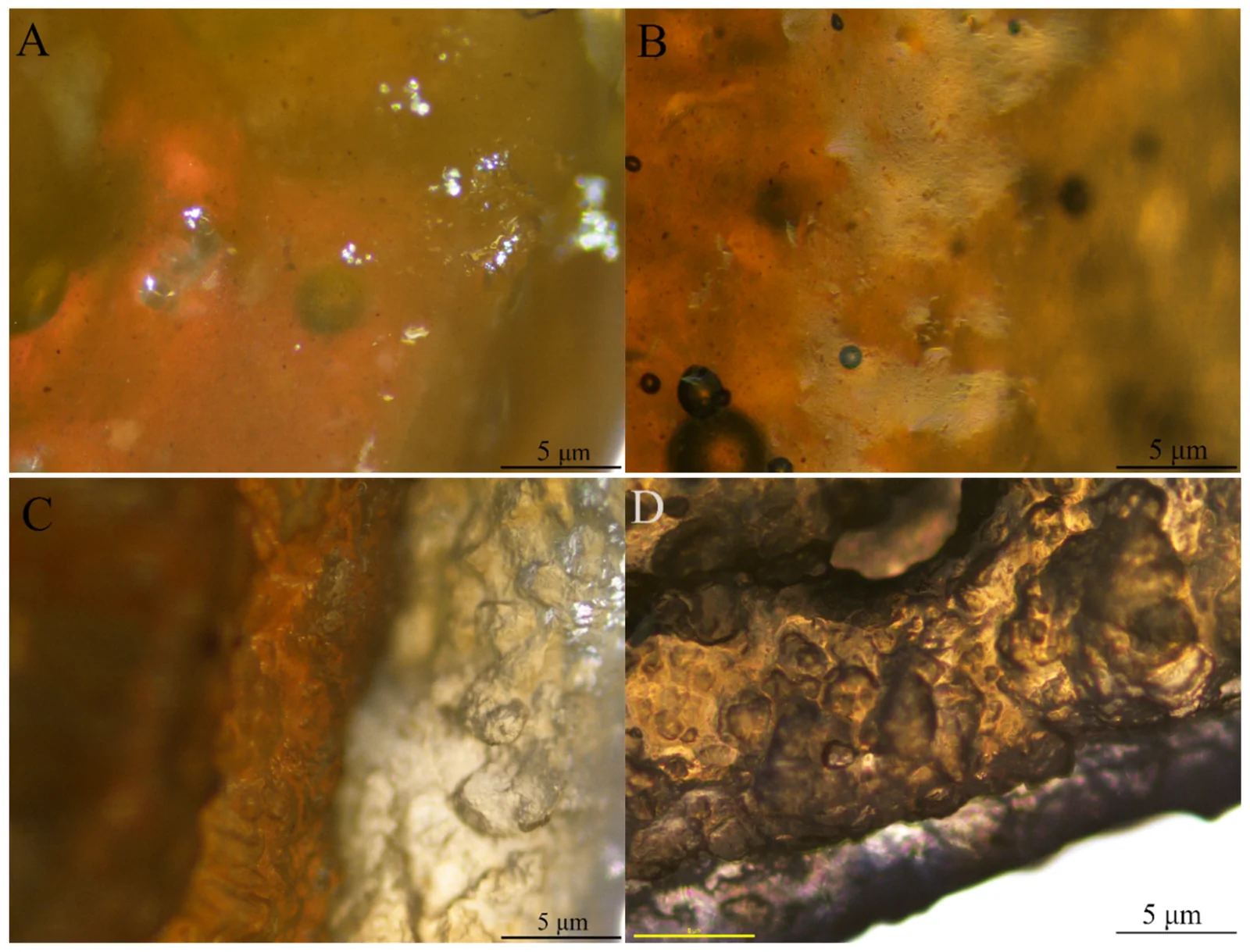
Visual analysis of the surface structure and texture via optical microscope at 4× magnification: experimental group gelatine–pectin printlet surface texture (**A**,**B**) and the layered structure (**C**,**D**).

The surface morphology of the printlet is a crucial factor in its functionality as a drug delivery platform since it significantly impacts the rate of water permeation and drug-release behaviour. Optical microscopy was specifically employed to assess the surface morphology and layer architecture of the printlets. As shown in Figure 10C,D, the printed layers of test printlets appear uniform and tightly bound, indicating good inter-layer adhesion and structural integrity of the gel-based formulation. It is seen that the printlets contain small bubbles, rough edges and small pores (Figure 10A,B); this is likely due to the nature of the polymers, polysaccharides, the flavones and CBD present [66]. However, the surface texture appears uneven, wavy, often times with visible pores. These features are consistent with entrapped air during extrusion. Similar surface roughness, porosity, and waviness have been reported for 3D-printed polysaccharide-based hydrogels when examined by optical and electron microscopy, where pore architecture and swelling behaviour are closely linked to microstructural heterogeneity [67].

**Figure 11:**
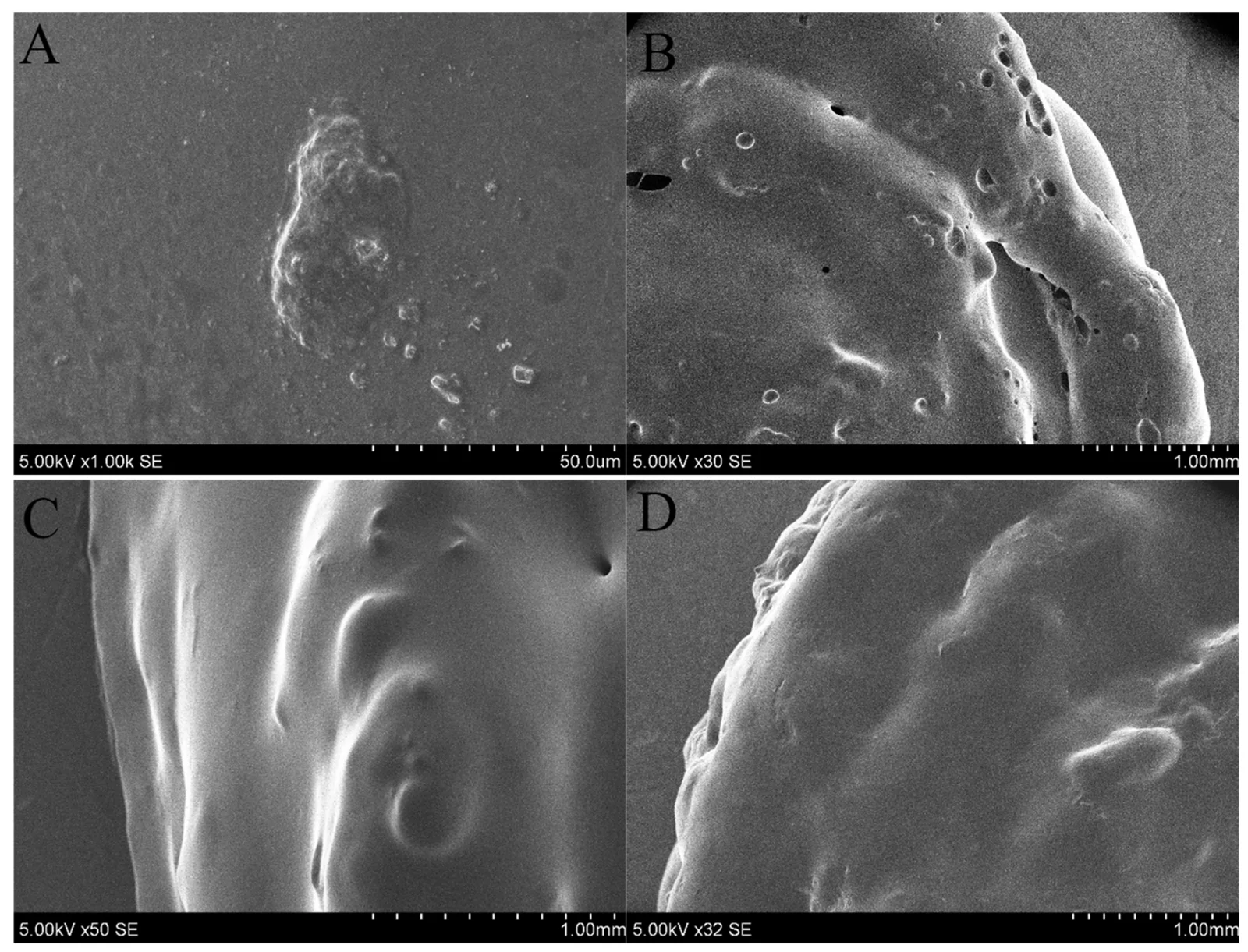
The visual representation via SEM analysis of the API crystals incorporated in the printlet (**A**), the porosity and its distribution of the experimental group gelatine–pectin printlets (**B**), and the visible layered structure (**C**,**D**) of the experimental group gelatine–pectin printlets.

SEM was further employed to confirm the layer architecture of the printlets (Figure 10C,D). The SEM images (Figure 11A) revealed the presence of small distributed API particles on the surface, along with distinct layered patterns characteristic of the 3D-printing process. Although some layering could be observed using optical microscopy and SEM, these features were not well-defined. Therefore, additional µ-CT analysis was conducted to achieve clearer visualisation and confirmation of the internal layer structure of the printlets.

Furthermore, SEM analysis provided detailed insights into the surface and microstructural features. The printlets exhibited a relatively coarse and rough surface morphology, along with compact porous structures that were largely closed and showed limited interconnectivity (Figure 11B). The pore size and shape within the printlet matrix are critical parameters, as they directly influence molecular transport and drug release [56,68]. Notably, these observations suggest the formation of a highly structured porous network in the bicontinuous system, particularly in the gelatine–pectin polymeric pair, indicating its potential role in modulating drug-release behaviour.

### 3.10. The Porosity and Pore Distribution Analysis by Computed Microtomography (µ-CT)

Gelatine–pectin printlets were scanned using a µ-CT system. The analysis was performed to identify and quantify the geometrical parameters of air voids within the samples. T1 represents gelatine–pectin control printlets and T2 the gelatine–pectin experimental group.

For each detected void, the volume, surface area, and coordinates of the centre of mass were calculated. Objects consisting of fewer than nine voxels were excluded from further analysis, as they were considered unreliable due to limited spatial resolution and segmentation uncertainty. The quantitative parameters determined by analysing 3D data are summarised in Table 5.

Void volume and surface area are commonly used parameters for describing pore size and morphology. However, to provide a more interpretable measure of void shape, the sphericity (Ψ_i_) of each void was calculated as:

$$\Psi_{i}=\frac{a_{s}}{a_{i}}=\frac{\sqrt[{3}]{36\pi {V_{i}}^{2}}}{a_{i}}$$

where Ψ*_i_* is the sphericity of the void, i; a_s_ is the surface area of the sphere, which has the same volume as the analysed void; a_i_ is the actual surface area of the void; and V_i_ is the volume of the void.

Sphericity equals to 1 for a perfect sphere, whereas lower values indicate less-compact objects. Due to the discrete nature of voxel-based reconstruction, surface area estimation becomes less accurate for small objects composed of only a few voxels. This may result in calculated sphericity values greater than 1. Therefore, values exceeding 1 were attributed to numerical artifacts and were set equal to 1.

Mean sphericity value for each sample was calculated as:

$$\Psi =\frac{1}{n}\sum_{i=1}^{n}\Psi_{i}$$

To account for discretisation effects inherent to voxel-based image analysis, the following correction was applied:

$$\Psi_{i}=\left\{\begin{array}{c} 1,\;if\;\;\left(\Psi_{i}>1\right)\;\\ \;\;\Psi_{i},\;if\;\;\left(\Psi_{i}\leq 1\right)\; \end{array}\right.$$

**Figure 12:**
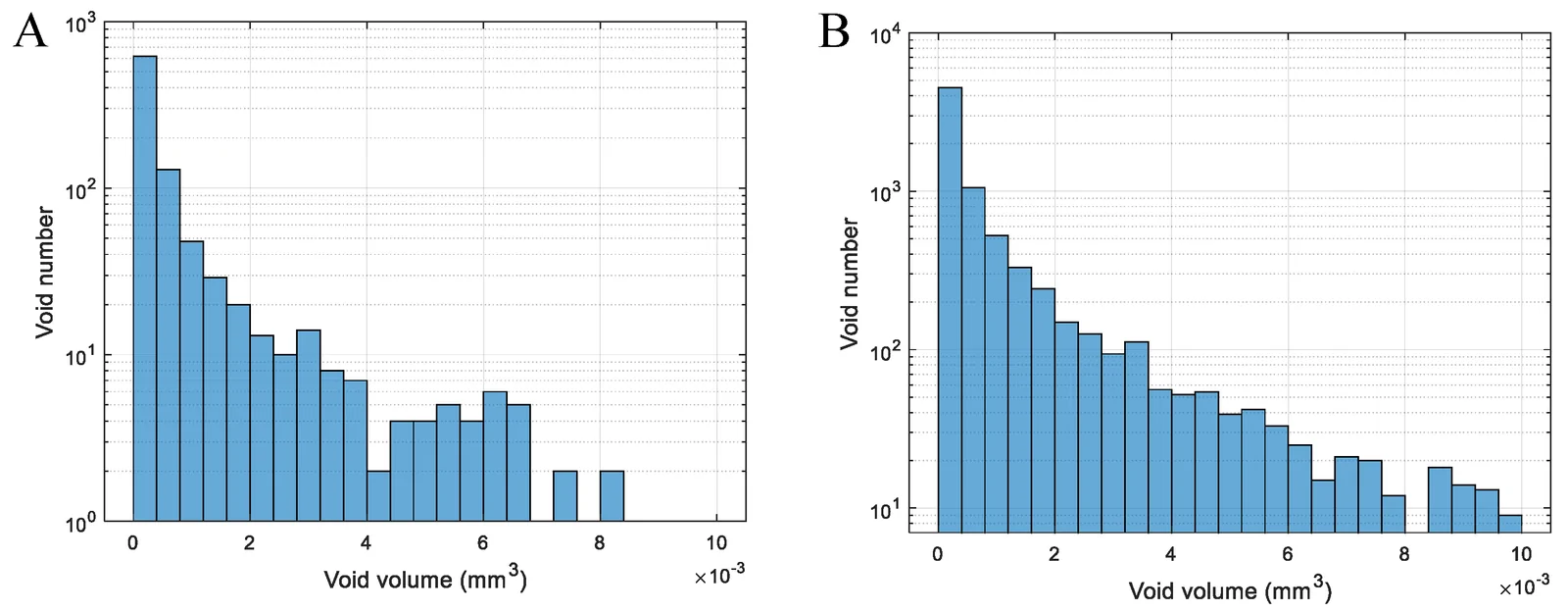
Histogram of void size distribution in (**A**) gelatine–pectin control printlets and (**B**) gelatine–pectin experimental group.

**Figure 13:**
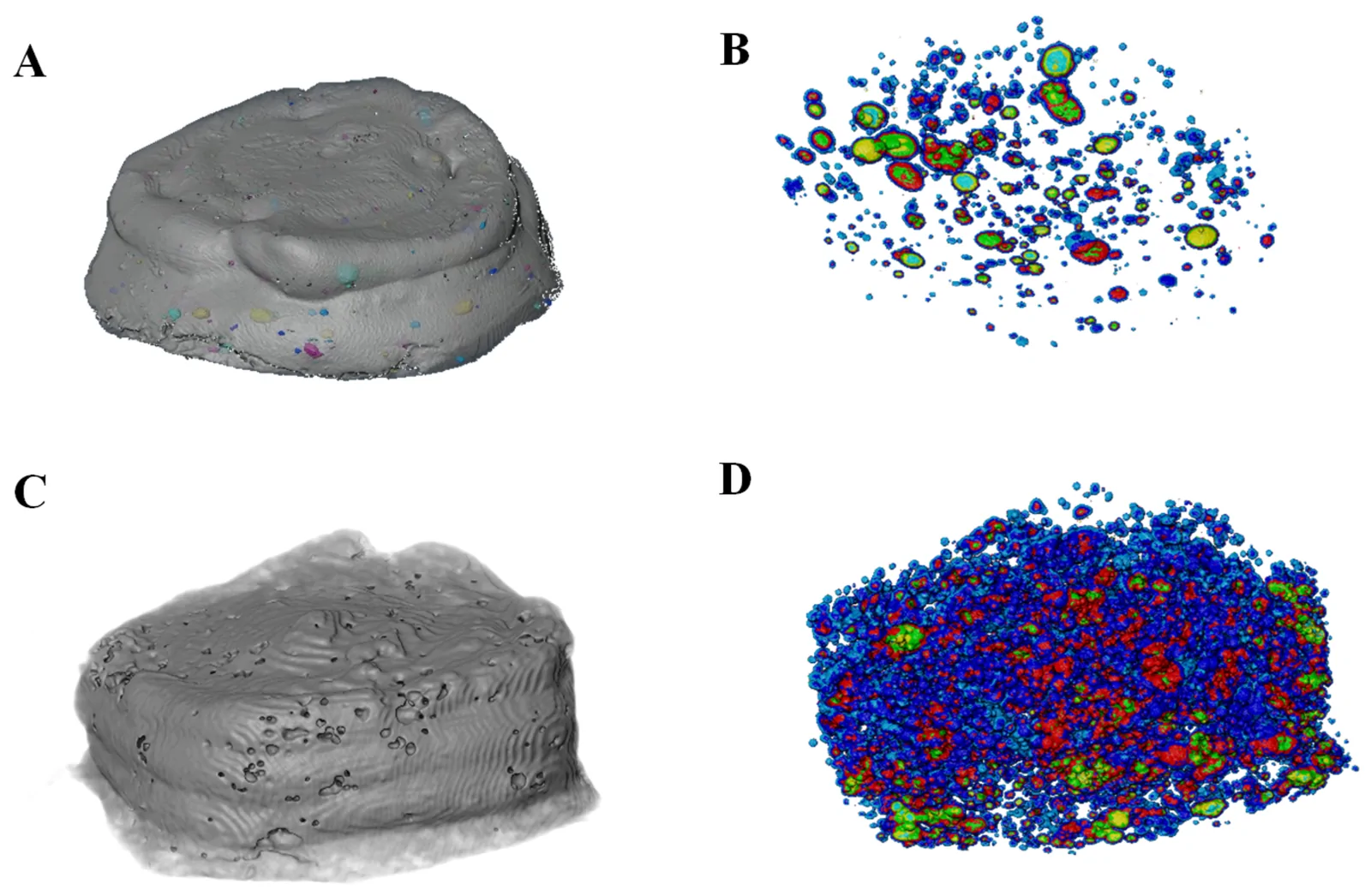
Visual 3D µ-CT images of the printlets: (**A**) T1 gelatine–pectin control printlet external visual, (**B**) the distribution of voids and pores in the gelatine–pectin control printlet, (**C**) T2 gelatine–pectin experimental printlet external visual, (**D**) the distribution of voids and pores in the gelatine–pectin experimental printlet.

The void size distributions in the printlets are presented in Figure 12 and Figure 13 and reveal distinct differences in internal microarchitecture for the control vs. experimental gelatine–pectin printlets. The results indicate that the control gelatine–pectin printlet exhibited fewer voids, and the voids are generally smaller in size compared with those observed in the gelatine–pectin experimental group printlet. Notably, even with the same gelatine–pectin polymeric pair, the control printlet demonstrated significantly reduced porosity relative to their API-loaded gelatine–pectin printlet. This increase in void content in the experimental group can be attributed to the incorporation of plant-based APIs, including apigenin, luteolin, and CBD. These components likely act as plasticizing agents within the polymeric matrix, reducing intermolecular interactions and structural cohesion during the printing and drying processes [69]. As a consequence, the matrix becomes more prone to pore formation and expansion, leading to increased void size and distribution (Figure 13). This observation is consistent with previous findings in polymer–API systems, where formulation composition plays a critical role in governing porosity and structural integrity. For instance, HPMC–PVP systems containing small-molecule APIs such as paracetamol or tranexamic acid have been shown to exhibit sensitivity to drying-induced porosity [70].

Shown in Figure 13 are two printlets of the gelatine–pectin control and gelatine–pectin experimental group visualised via X-ray µCT. The chemical nature of the API can significantly interfere with intermolecular bonding between polymers. Incorporation of APIs into gelatine–pectin matrices significantly increased pore size and porosity compared to the API-free control, as observed via microstructural analysis (Figure 13). This structural expansion is consistent with weaker polymer–API interactions in the gelatine–pectin complex, evidenced by lower docking scores (−4.18 to −4.99 kcal/mol) and an absence of H-bonding with apigenin, luteolin, and CBD. Consequently, the looser network facilitates faster API diffusion, favouring immediate-release profiles over the denser structures in controls or alternative polymer pairs like agar–pectin. Similar behaviour has been reported in protein–polyphenol systems; the addition of salicylic acid significantly lowers the gelation temperature because its acidic nature disrupts the crosslinking interactions [71]. Such disruptions can lead to a less stable internal network, potentially increasing the size or irregularity of the pores. The porous structure of the gelling matrix is a key factor in the controlled release of flavones [72]. This behaviour is corroborated with molecular docking of the gelatine–pectin pair with APIs, seeing the lowest docking score with no bonding compared to other polymeric pairs.

### 3.11. Disintegration Test

Disintegration is a pivotal stage in the dissolution process of solid oral dosage forms, influencing both the onset and rate of drug release. In this study, the disintegration time of various moulded tablets and printlets was evaluated in gastric and intestinal pH environments, following pharmacopeial standards. The results showed a wide variation in disintegration, ranging from 15 to 35 min depending on the formulation and medium (Table 6).

The HPMC–gelatine samples demonstrated effective disintegration in both gastric and intestinal fluids. Within this group, the printlets disintegrated within about 30 min, whereas the conventionally moulded tablets disintegrated more rapidly, within about 20 min (Table 6). This difference can be attributed to the physical characteristics imparted by the manufacturing method. Moulded tablets tend to be softer and more uniform, enabling faster fluid penetration and matrix breakdown. In contrast, printlets exhibited a firmer texture with slight tackiness and a distinct layered architecture absent in moulded tablets. This layered structure likely affects moisture retention, tablet texture, and consequently slows disintegration and drug release kinetics by limiting fluid ingress into the matrix [73,74].

Other formulations, including those composed of gelatine–pectin, did not exhibited complete disintegration in gastric fluid within 30 min, necessitating further evaluation in intestinal fluid. Here, the control group’s gelatine–pectin printlets disintegrated within 30 min, whereas moulded tablets disintegrated faster, within 15 min. Similarly, within the experimental group, moulded tablets disintegrated at 15 min, but the printlets showed the longest disintegration time of 35 min (Table 6). These differences again relate to differences in tablet density and microstructure induced by 3D printing, which creates a more compact matrix, hindering fluid penetration [75,76].

These behaviours can be further explained by the intrinsic properties of the polymers used. Gelatine, in its non-crosslinked form, is highly biodegradable and tends to disintegrate rapidly, contributing to shorter disintegration times [27,77]. In contrast, pectin exhibits strong pH-dependent behaviour: in acidic media, it forms a dense, rubbery gel layer that limits hydration and delays disintegration, whereas in neutral or slightly alkaline conditions, it swells more extensively, promoting faster matrix breakdown [78,79,80]. This explains the improved disintegration observed in buffer conditions. Agar-based systems form stiffer and more crystalline gel networks, which can resist rapid disintegration due to reduced swelling capacity [81,82]. Meanwhile, HPMC rapidly hydrates to form a viscous gel barrier that can delay erosion; however, when combined with gelatine, the overall system tends toward faster disintegration due to gelatine’s dominant behaviour.

In summary, differences in disintegration times across formulations and manufacturing methods stem from variations in tablet texture, microstructure, polymer composition, and pH-responsive behaviour and moisture retention capabilities. Moulded tablets tend to be more porous and softer, leading to faster disintegration, whereas the firmer, layered architecture of printlets may retard fluid ingress and slow disintegration. Polymer type and environment (gastric vs. intestinal pH) further modulate swelling and solubility properties influencing disintegration kinetics. Understanding these factors is critical for optimising tablets to achieve desired drug-release profiles.

### 3.12. In Vitro Dissolution of Moulded Tablets and Printlets

Dissolution behaviour of moulded tablets and printlets was evaluated in simulated gastric conditions (pH 1.2) for 90 min, followed by transfer to the simulated intestine conditions (pH 6.8). The dissolution study was continued until complete disintegration and drug release from dosage form. Most tablets and printlets exhibited dissolution within ~180 min in total (90 min in pH 6.8); therefore, it was decided to keep the time margin equal for all the samples for an easier and clearer comparison. The complete dissolution time varied between 180 and 210 min, where gelatine–pectin (180 min) and HPMC–gelatine (30–60 min) tablets were dissolved completely, and agar–pectin and HPMC–pectin were dissolved completely in 200–210 min.

**Figure 14:**
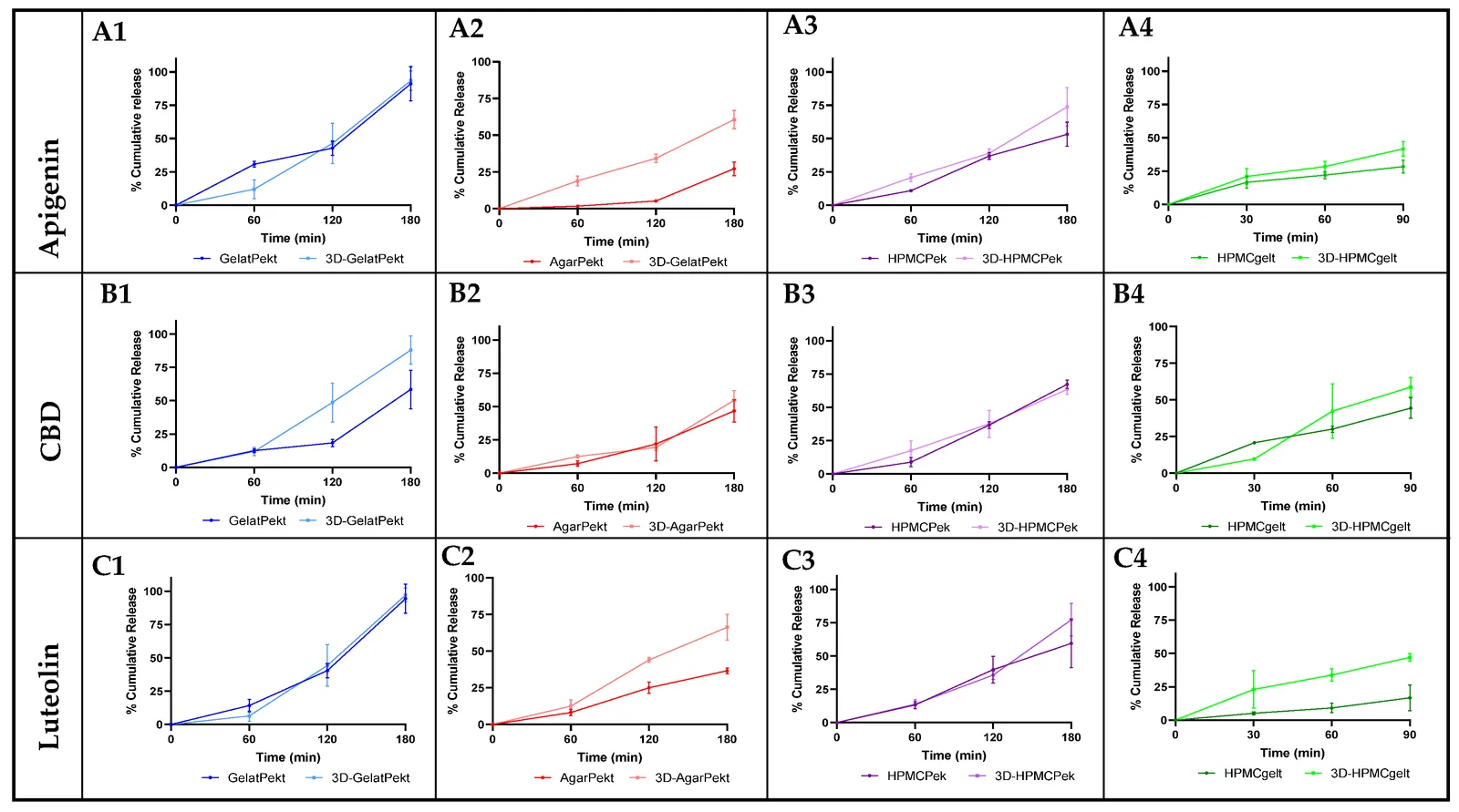
In vitro dissolution profiles of moulded tablets and printlets prepared using four different polymer pair systems: agar–pectin (AgarPekt), gelatine–pectin (GelatPekt), HPMC–gelatine (HPMCgelt), and HPMC–pectin (HPMCPek). Panels (**A1**–**A4**) show apigenin release from (**A1**) GelatPekt, (**A2**) AgarPekt, (**A3**) HPMCPek, and (**A4**) HPMCgelt tablets. Panels (**B1**–**B4**) show CBD release from (**B1**) GelatPekt, (**B2**) AgarPekt, (**B3**) HPMCPek, and (**B4**) HPMCgelt tablets. Panels (**C1**–**C4**) show luteolin release from (**C1**) GelatPekt, (**C2**) AgarPekt, (**C3**) HPMCPek, and (**C4**) HPMCgelt tablets. Data are presented as mean ± SD (*n* = 3).

In the intestinal pH 6.8, the polymers undergo increased ionisation, which enhances electrostatic repulsion among polymer chains. This results in greater swelling, and relaxation of the polymeric matrix structure. These structural changes facilitate faster diffusion of the API, explaining the significant increase in drug release observed in Figure 14. This behaviour was more evident in the gelatine-containing moulded tablets, and printlets demonstrated a clear pH-dependent release. Under acidic conditions, gelatine carries a positive charged (pH < isoelectric point) and forms a compact gel structure through electrostatic interactions with co-polymers and the API, thereby restricting limiting swelling and molecular diffusion. This behaviour demonstrated the influence of pH-responsive polymer behaviour in controlling drug release. Similar trends have been reported for gelatine-based hydrogels and capsule systems, where an accelerated drug release is observed at neutral to alkaline pH due to increased polymer ionisation, swelling and partial dissolution [83,84,85].

The dissolution profiles revealed significant differences in release behaviour across the polymer systems and fabrication methods (Figure 14). Among all formulations, the highest API release was observed in the gelatine–pectin printlets, with luteolin showing the greatest release (97.25 ± 5.25%), followed by apigenin (93.45 ± 7.21%) and CBD (87.98 ± 10.55%). In comparison, the corresponding moulded tablets exhibited a notably lower release for CBD (58.33 ± 14.40%), while apigenin (91.08 ± 12.85%) and luteolin (94.50 ± 10.95%) showed slightly reduced but still high release. Gelatine–pectin formulations are especially an interesting case. The higher release among the systems was obtained by gelatine–pectin tablets, demonstrating their applicability for compositions requiring immediate release. In most studies this classic combination is expected to have an immediate release profile [27,86]. Gelatine tends to readily dissolve or disperse upon exposure to warm aqueous media. This behaviour is also closely related to its gelling properties and mechanical strength, as gelatine forms a thermally reversible gel. The rigidity and hardness of gelatine-based formulations are directly proportional to the gelatine concentration, and these factors significantly influence the dissolution behaviour of both tablets and printlets [87,88]. In this study, formulations F1 and F5 contained higher gelatine content (8.43–9.40% *w*/*w*), whereas F3 and F7 contained lower amounts (6.28–7.00% *w*/*w*). This difference in composition likely contributed to variations in dissolution behaviour, with F1 and F5 exhibiting slower drug release and longer dissolution times compared to F3 and F7 (Figure 14).

Pectin also plays a crucial role in governing release kinetics. As discussed previously, its behaviour is highly pH-dependent in acidic media; pectin exhibits reduced solubility, whereas in neutral to alkaline conditions (pH 6.8–7.4), it undergoes extensive swelling. This swelling behaviour contributes to delayed drug release in the gastric environment, followed by a rapid or pulsatile release upon transition to intestinal conditions, making pectin a promising carrier for site-specific drug delivery [89,90]. Pectin matrix tablets typically form a continuous gel layer upon contact with aqueous media, and their subsequent breakdown is influenced by a combination of swelling and erosion. This mechanism dictates that API release kinetics usually fit the Korsmeyer–Peppas equation [91]. This behaviour explains the sharp increase in release observed for all three APIs at 120 min.

In the HPMC–pectin formulations, printlets exhibited higher release for apigenin (73.79 ± 14.45%) and luteolin (77.25 ± 12.27%) compared to moulded tablets (53.20 ± 8.96% and 59.46 ± 18.19%, respectively), while CBD release was slightly higher in moulded tablets (67.20 ± 3.16%) than in printlets (63.44 ± 3.80%). Conversely, the HPMC–gelatine system showed overall lower release compared to other formulations. The printlets achieved CBD release of 59.00 ± 6.49%, apigenin 42.00 ± 5.73%, and luteolin 47.00 ± 2.81%, whereas the moulded tablets exhibited further reduced release, particularly for luteolin (17.00 ± 9.70%), with CBD at 45.00 ± 8.03% and apigenin at 29.00 ± 5.04%. Markedly, printlets displayed higher API release than their moulded method, attributed to the increased surface area and internal micro-voids arising from the layered printing structure.

These formulation-dependent differences can be mechanistically attributed to the hydration and swelling behaviour of HPMC and its interaction with secondary polymers, as well as to structural differences arising from the manufacturing method. HPMC is a hydrophilic polymer that rapidly hydrates and swells upon contact with water; therefore, its presence can control drug-release kinetics. In certain literature, it is observed that when combining two gelling agents, for instance gelatine and carrageenan, the tablets delivered rapid dissolution (>80–85% in 30 min), suggesting immediate release and good patient centricity [70,92]. The combination of HPMC–gelatine is expected to be chewed and dissolved rapidly at 37 °C, with the drug being typically released within the first 15 min [93]. Therefore, the HPMC–gelatine formulation tablets and printlets were dissolved in the 30–60 min timespan in the gastric environment.

In contrast, agar–pectin formulations, irrespective of the fabrication method, consistently exhibited the lowest phytochemical release (Figure 14), indicating the formation of a dense and highly resistant gel matrix. The printlets demonstrated moderate release with luteolin (66.32 ± 8.81%), apigenin (60.63 ± 6.14%), and CBD (54.66 ± 7.17%), whereas the moulded tablets showed considerably lower release, particularly for apigenin (27.10 ± 4.67%) and luteolin (36.41 ± 2.00%), with CBD at 46.73 ± 8.40%. Although pure agar is readily dispersible, its incorporation into composite matrices (e.g., with pectin or cellulose) promotes the development of a more ordered and crystalline network, resulting in increased matrix density and reduced swelling capacity [57]. This structural reinforcement limits water penetration and significantly retards drug diffusion. This mechanistic interplay explains the comparatively slower release observed in agar–pectin tablets and printlets relative to the gelatine–pectin system. Specifically, agar enhances matrix rigidity and diffusion resistance, while pectin contributes a pH-responsive barrier forming a compact surface layer under acidic conditions that further limits fluid ingress and prolongs drug release.

The Korsmeyer–Peppas model provided the best fit for the release profiles of CBD, apigenin, and luteolin from both moulded tablets and printlets, as evidenced by high R^2^ values (Supplementary Tables S1–S6). The transport exponent (*n*) values obtained for HPMC–gelatine formulations in both moulded tablets and printlets were ≤1, indicating non Fickian (anomalous) transport. This suggests that drug release is governed by a combination of diffusion and polymer relaxation/swelling mechanisms, resulting in sustained API release. However, CBD-loaded HPMC–gelatine printlets and luteolin-loaded HPMC–gelatine moulded tablets exhibited *n* values close to or slightly greater than unity (*n* ≈ 1.1–1.3), indicating a transition towards Case II or Super Case II transport behaviour. In contrast, tablets and printlets containing other polymer combinations exhibited *n* > 1, characteristic of Super Case II transport, where drug release is predominantly controlled by extensive polymer swelling, matrix erosion, or restructuring of the polymer network. This behaviour correlates well with the swelling characteristics of polymers observed in intestinal medium (Figure 14), highlighting their influence on the release mechanism.

Nevertheless, the enhanced drug release at pH 6.8, attributed to the pH-responsive swelling and relaxation behaviour of gelatine under intestinal conditions, suggests that these formulations can be tailored for faster drug release and may be suitable for the development of immediate or targeted release polymeric delivery systems, depending on the polymer composition and environmental pH.

Furthermore, these findings also support evidence from the literature that strong intermolecular interactions, particularly hydrogen bonding, play a critical role in modulating release profiles. Stronger hydrogen bonding has been repeatedly shown to reduce diffusion and prolong release by stabilising drug–polymer complexes and limiting molecular mobility [94,95,96,97]. In the present study, a clear relationship was observed between stronger binding energies and slower release, exemplified by the agar–pectin system, indicating strong polymer–polymer and polymer–API interactions. In contrast, gelatine–pectin pairs exhibited the fastest release, which may be advantageous for immediate-release applications, whereas HPMC-based formulations provided intermediate profiles.

Overall, the results confirmed that the potential of polymer–polymer and polymer–API interactions played a decisive role in governing matrix integrity and drug-release behaviour. Stronger interactions, such as agar–pectin, provide enhanced stability and controlled release, whereas weaker binding, such as gelatine–pectin, facilitates complete and faster release. The integrated interpretation of in silico modelling and experimental dissolution outcomes provides a rational approach for excipient selection and optimisation of gel-based tablets with tailored release profiles.

### 3.13. Stability of Moulded Tablets and Printlets

The samples of printlets and moulded tablets were placed in the stability chamber at 25 °C/60% (RH) and were collected and monitored at time stamps of 1, 3 and 6 months. After 1 and 3 months the printlets and regular tablets remained stable, demonstrating similar or same results, with only slight changes; however, after 6 months, after conducting all of the assays, the changes in qualitive and quantitative properties occurred.

When conducting moisture and texture (hardness and stickiness) analysis as well as the disintegration and dissolution assays, both tablets and printlets after 1 and 3 months showed no signs of change or just minor changes, but after 6 months, the moisture content had greatly reduced, affecting the texture; hardness had increased, stickiness had decreased, the disintegration and dissolution time had prolonged, and the API content had diminished (3–4 times in printlets, and for moulded tablets it was by 2–4 times, in all excipient formulations). According to the SPSS related-samples Wilcoxon signed-rank test, the characteristics and the API content had statistically significantly (*p* < 0.05) changed over the course of 6 months, but not 1 to 3 months.

The results show that both conventional tablets and printlets lose their key characteristics and the API content after 6 months in the stability chamber, which concludes that they are intended for extemporal use, meaning that they are intended for immediate and direct consumption after being produced fresh.

This study successfully demonstrated the feasibility and advantages of 3D printing for gel-based tablet formulation. However, certain restrictions, opportunities for development, and areas for future research remain when compared with traditionally prepared moulded tablets. The work highlights the potential of SSE-3D printing for formulating personalised dosage forms, emphasizing the importance of molecular interactions between excipients, which are crucial for tailoring release profiles. This knowledge is important to the successful translation of 3D-printed personalised dosage forms into clinical practice.

## 4. Conclusions

This work successfully demonstrates the molecular modelling of interactions between polymers and plant-derived APIs can help predict formulation behaviour and differences in drug-release profiles from rationally designed moulded tablets and SSE 3D-printed printlets. The study systematically compared conventional moulding and SSE 3D printing, with particular emphasis on molecular interactions, rheological properties, internal microstructure, pharmaceutical performance, and drug-release behaviour. It also demonstrated the successful incorporation of *Artemisia annua* L. extract, represented by apigenin and luteolin, together with CBD into gel-based printlets containing different polymer combinations. The moulded tablet and SSE printlets formulated with different polymer pairs further demonstrated superior hardness, stickiness, moisture content, and phytochemical API release compared with moulded tablets.

The most significant finding was the agreement between the molecular modelling predictions and experimental results. Agar–pectin exhibited the strongest polymer–polymer and polymer–API interactions, including multiple hydrogen bonds, and formed a dense matrix associated with slower API release. In contrast, gelatine–pectin displayed weaker interactions and no hydrogen bonding with the selected APIs in the modelled poses, producing a less cohesive and more porous structure that facilitated faster release (luteolin—97.25 ± 5.25%, apigenin—93.45 ± 7.21%, and CBD—87.98 ± 10.55%). Micro-CT analysis provided important structural confirmation, and API incorporation increased the void volume of gelatine–pectin printlets from 1.15% to 8.77%, demonstrating that disruption of polymer interactions contributed to pore formation and enhanced molecular diffusion.

Gelatine–pectin offered the most favourable overall balance of rapid solidification, printability, mechanical strength, manageable stickiness, porosity, and API release, supporting its suitability for immediate-release printlets. Conversely, the dense agar–pectin network showed potential for formulations requiring slower or prolonged release. The layered architecture generated by SSE printing also affected moisture retention, hardness, disintegration, and dissolution relative to moulded tablets. Overall, the molecular–mechanistic correlations established in this study provide a rational design pathway for selecting excipients and developing next-generation, nature-inspired, personalised 3D-printed drug-delivery systems with tailored release characteristics.

## Figures and Tables

**Table 1:** The amounts % (*w/w*) of the excipients used for different formulations.

Formulation	Gelatine (%)	Agar (%)	Hydroxypropyl-Methylcellulose (%)	Pectin (%)	Sugar (%)	Citric Acid (%)	Water (%)	*Artemisia annua* L. Extract (%)	CBD (%)
F1	9.40	–	–	7.50	26.30	0.50	56.30	–	–
F2	–	9.40	–	7.50	26.30	0.50	56.30	–	–
F3	7.00	–	1.40	–	26.30	0.50	64.80	–	–
F4	–	–	1.40	7.50	26.30	0.50	64.30	–	–
F5	8.43	–	–	6.73	23.59	0.45	50.50	10	0.3
F6	–	8.43	–	6.73	23.59	0.45	50.50	10	0.3
F7	6.28	–	1.26	–	23.59	0.45	58.12	10	0.3
F8	–	–	1.26	6.73	23.59	0.45	57.67	10	0.3

**Table 2:** Molecular modelling generated best pose values of binding energy and H-bond energy of polymer–polymer systems.

Polymer 1	Polymer 2	Binding Energy (kcal/mol)	H-Bond Energy (kcal/mol)
Agar	Pectin	−8.72	−0.5, −1.6, −3.6, −3.8
HPMC	Pectin	−6.32	−1.5, −1.5, −3.3
Gelatine	Pectin	−6.23	-
HPMC	Gelatine	−7.25	−2.8, −2.3, −1.5

**Table 3:** Molecular modelling generated best pose values of binding energy and H-bond energy of polymer complexes–API systems.

Polymer Complex	API	Binding Energy (kcal/mol)	H-Bond Energy (kcal/mol)
Agar–pectin complex	Apigenin	−5.81	−1.8, −2.1, −3.7
HPMC–pectin complex		−4.79	-
Gelatine–pectin complex		−4.18	-
HPMC–gelatine complex		−5.15	-
Agar–pectin complex	CBD	−5.29	−0.5
HPMC–pectin complex		−5.15	-
Gelatine–pectin complex		−4.99	-
HPMC–gelatine complex		−4.84	-
Agar–pectin complex	Luteolin	−5.88	−1.6, −2.1
HPMC–pectin complex		−4.74	-
Gelatine–pectin complex		−4.47	-
HPMC–gelatine complex		−4.86	-

**Table 4:** The uniformity of mass results and the deviation of tablet and printlet samples (*n* = 20).

Formulation	Group	Dosage Form	Mass (mg)
Gelatine–pectin	Control	Moulded tablet	908.7 ± 4.1
		Printlet	837.5 ± 3.7
	Experimental	Moulded tablet	923.4 ± 6.7
		Printlet	857.6 ± 1.2
Agar–pectin	Control	Moulded tablet	957.3 ± 1.7
		Printlet	883.3 ± 4.3
	Experimental	Moulded tablet	962.3 ± 3.5
		Printlet	878.5 ± 4.5
HPMC–Gelatine	Control	Moulded tablet	1003.1 ± 4.7
		Printlet	871.5 ± 3.9
	Experimental	Moulded tablet	1092.7 ± 6.3
		Printlet	879.8 ± 3.7
HPMC–Pectin	Control	Moulded tablet	996.5 ± 3.9
		Printlet	895.8 ± 3.4
	Experimental	Moulded tablet	1152.6 ± 6.1
		Printlet	892.8 ± 3.4

**Table 5:** Void measurement parameters in printlets.

Sample Code	T1	T2
Sample volume, mm^3^	145.78	166.99
Void volume, mm^3^	1.68	14.64
Void volume percentage, %	1.15	8.77
Void mean size, mm^3^	1.76 × 10^−3^	1.88 × 10^−3^
Void size median, mm^3^	16 × 10^−5^	27 × 10^−5^
Void count	956	7805
Void density, pcs/mm^3^	6.56	46.74
Sample surface area, mm^2^	243.9	281.6
Void surface area, mm^2^	44.1	483.5
Mean sphericity	0.96	0.95

**Table 6:** Comparison of disintegration times of moulded tablets vs. printlets.

Formulation	Control Moulded Tablet (min)	API Moulded Tablet (min)	Control Printlets (min)	API Printlets (min)
**Gelatine–Pectin**	15	15	35	30
**Agar–Pectin**	30	15	30	15
**HPMC–Gelatine**	20	20	30	30
**HPMC–Pectin**	35	30	35	30

## Data Availability

The data presented in this study are available on request from the corresponding author.

## Supplementary Materials

The following supporting information can be downloaded at https://www.mdpi.com/article/10.3390/pharmaceutics18081035/s1, Figure S1: Comparative evaluation between the alcoholic and dealcoholized extracts; Figure S2: Representation of the X-ray computed tomography setup used; Figure S3: Photograph of the experimental setup. Table S1: CBD release kinetic of printlets; Table S2: Apigenin release kinetic of printlets; Table S3: Luteolin release kinetic of printlets; Table S4: CBD release kinetic of moulded tablets; Table S5: Apigenin release kinetic of moulded tablets; Table S6: Luteolin release kinetic of moulded tablets.
