# Supplementary material for: Mechanistic Comparison of Semi-Solid Extrusion 3D-Printed Printlets and Hot-Moulded Tablets: Linking Polymer–API Interactions, Microstructure, and Dissolution of Plant-Based Formulations
Source: Pharmaceutics. 2026 Aug 20;18(8):1035. doi: 10.3390/pharmaceutics18081035 (PMC13516868; doi:10.3390/pharmaceutics18081035)
Supplement: Supplementary file 1 [file pharmaceutics-18-01035-s001.zip › pharmaceutics-4457095-supplementary.pdf]

1. Drug release kinetic models

Table S1 CBD release kinetic of printlets

CBD Release Kinetic					
**Release Kinetics**		**3D-GelatPekt**	**3D-AgarPekt**	**3D-HPMCgelt**	**3D-HPMCPek**
**Zero order (ZO) R^2**		0.913	0.871	0.919	0.944
**First order (FO) R^2**		0.815	0.821	0.881	0.913
**Higuchi (HG) R^2**		0.732	0.701	0.771	0.833
**Hixson (HX) (R^2)**		0.847	0.837	0.896	0.926
**Korsemeyer Peppas KP**	**R^2**	0.971	0.939	0.9225	0.956
	**n**	1.68	2.00	1.362	1.23
**Best fit model**		**KP**	**KP**	**KP**	**ZO/KP**

Table S2 Apigenin release kinetic of printlets

Apigenin Release Kinetic					
**Release Kinetics**		**3D-GelatPekt**	**3D-AgarPekt**	**3D-HPMCgelt**	**3D-HPMCPek**
**Zero order (ZO) R^2**		0.898	0.985	0.899	0.975
**First order (FO) R^2**		0.784	0.953	0.929	0.917
**Higuchi (HG) R^2**		0.708	0.875	0.944	0.845
**Hixson (HX) (R^2)**		0.819	0.965	0.921	0.937
**Korsemeyer Peppas KP**	**R^2**	0.965	0.984	0.984	0.985
	**n**	1.79	1.13	0.713	1.27
**Best fit model**		**KP**	**ZO/KP**	**KP**	**KP/ZO**

Table S3 Luteolin release kinetic of printlets

Luteolin Release Kinetic					
**Release Kinetics**		**3D-GelatPekt**	**3D-AgarPekt**	**3D-HPMCgelt**	**3D-HPMCPek**
**Zero order (ZO) R^2**		0.858	0.962	0.879	0.926
**First order (FO) R^2**		0.732	0.915	0.917	0.842
**Higuchi (HG) R^2**		0.649	0.817	0.916	0.743
**Hixson (HX) (R^2)**		0.770	0.934	0.907	0.869
**Korsemeyer Peppas KP**	**R^2**	0.989	0.971	0.971	0.991
	**n**	2.17	1.28	0.734	1.72
**Best fit model**		**KP**	**KP**	**KP**	**KP**

Table S4 CBD release kinetic of moulded tablets

CBD Release Kinetic					
**Release Kinetics**		**GelatPekt**	**AgarPekt**	**HPMCgelt**	**HPMCPek**
**Zero order (ZO) R^2**		0.844	0.865	0.9535	0.930
**First order (FO) R^2**		0.787	0.824	0.972	0.864
**Higuchi (HG) R^2**		0.668	0.685	0.965	0.744
**Hixson (HX) (R^2)**		0.805	0.838	0.968	0.887
**Korsemeyer Peppas KP**	**R^2**	0.934	0.943	0.989	0.989
	**n**	2.261	2.200	0.721	1.634
**Best fit model**		**KP**	**KP**	**KP**	**ZO / KP**

Table S5 Apigenin release kinetic of moulded tablets

Apigenin Release Kinetic					
**Release Kinetics**		**GelatPekt**	**AgarPekt**	**HPMCgelt**	**HPMCPek**
**Zero order (ZO) R^2**		0.936	0.704	0.86	0.950
**First order (FO) R^2**		0.865	0.678	0.897	0.923
**Higuchi (HG) R^2**		0.833	0.492	0.970	0.823
**Hixson (HX) (R^2)**		0.888	0.686	0.886	0.934
**Korsemeyer Peppas KP**	**R^2**	0.935	0.993	0.996	0.949
	**n**	1.251	3.970	0.511	1.208
**Best fit model**		**ZO & KP**	**KP**	**KP**	**ZO/KP**

Table S6 Luteolin release kinetic of moulded tablets

Luteolin Release Kinetic					
**Release Kinetics**		**GelatPekt**	**AgarPekt**	**HPMCgelt**	**HPMCPek**
**Zero order (ZO) R^2**		0.900	0.972	0.958	0.927
**First order (FO) R^2**		0.782	0.959	0.952	0.902
**Higuchi (HG) R^2**		0.707	0.847	0.879	0.824
**Hixson (HX) (R^2)**		0.817	0.964	0.954	0.912
**Korsemeyer Peppas KP**	**R^2**	0.993	0.980	0.985	0.948
	**n**	1.914	1.167	1.110	1.252
**Best fit model**		**KP**	**ZO/KP**	**KP**	**KP**

2. The extraction process of *Artemisia annua L. plant*

A comparative evaluation between the alcoholic (80% v/v) and dealcoholized extracts revealed notable differences in phytochemical content. The dealcoholized extract showed a substantially higher concentration of chlorogenic acid (3541.40 ± 177.070 µg/g) compared to the alcoholic extract (1287.764 ± 64.387 µg/g), corresponding to an approximately 2.75-fold increase. Similarly, neochlorogenic acid content was moderately higher in the dealcoholized extract (70.880 ± 3.544 µg/g) than in the alcoholic extract (49.469 ± 2.473 µg/g), representing an

increase of about 1.4-fold. In contrast, the concentrations of apigenin and luteolin were comparable between the two extracts, with only marginal differences (<2%), likely within experimental variability.

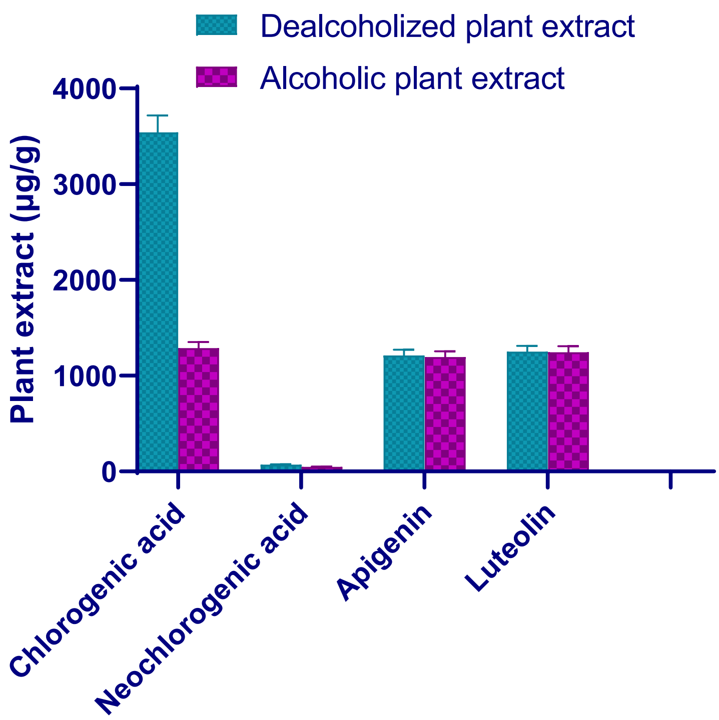

Figure S1 Comparative evaluation between the alcoholic and dealcoholized extracts

3. X-ray Computed Tomography

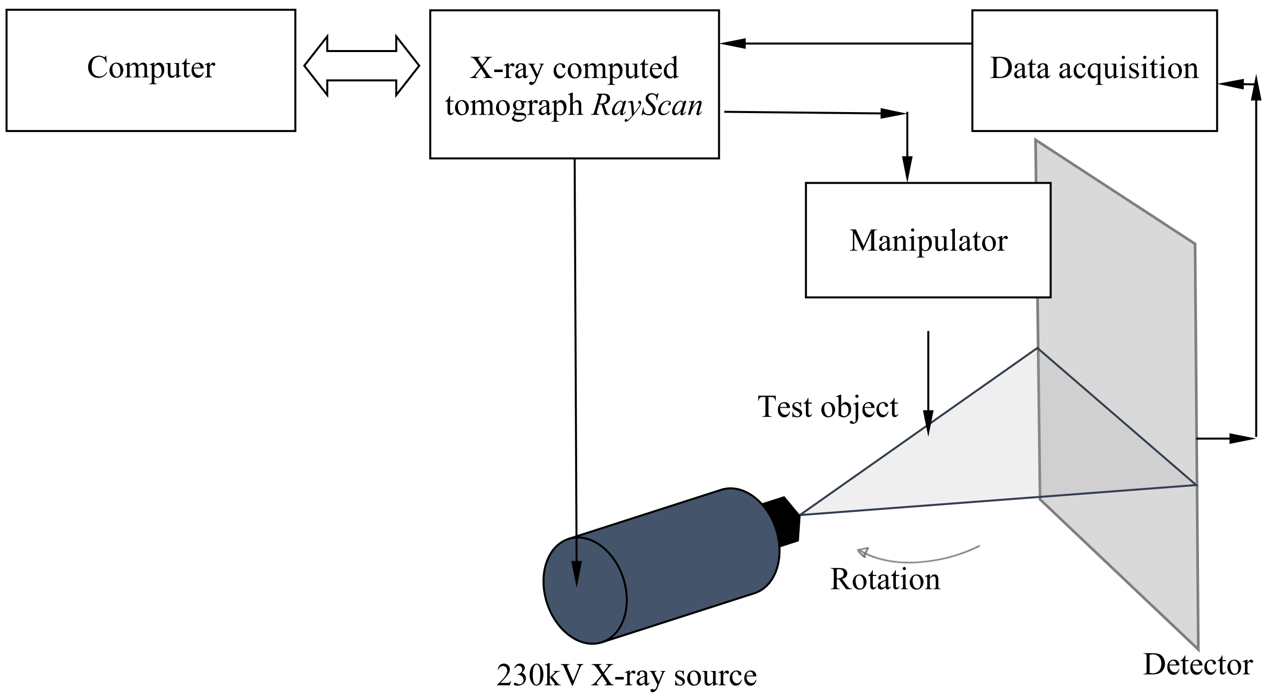

Figure S2 Representation of the X-ray computed tomography setup used

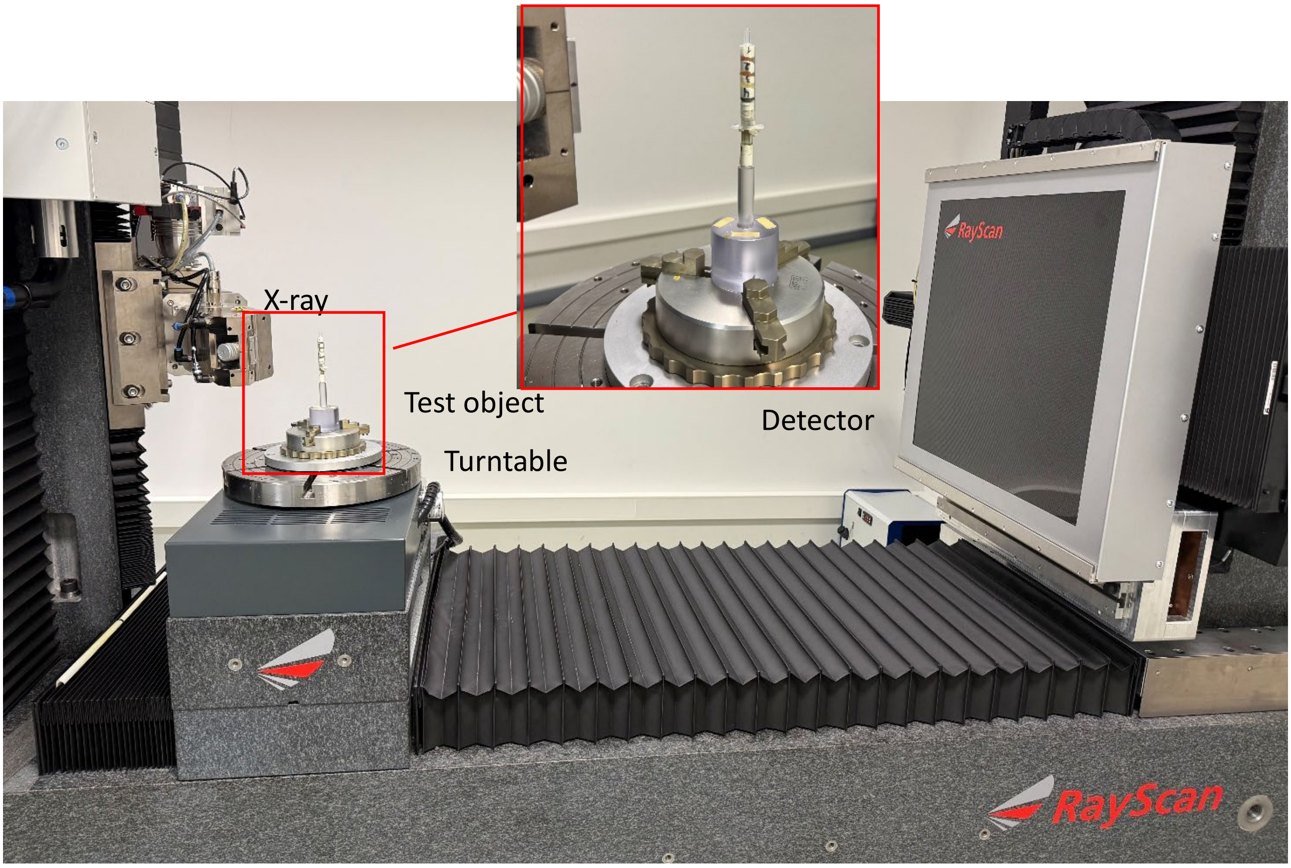

Figure S3 Photograph of the experimental setup
